# Skeletal muscle regeneration after extensive cryoinjury of caudal myomeres in adult zebrafish

**DOI:** 10.1038/s41536-024-00351-5

**Published:** 2024-02-20

**Authors:** Hendrik Oudhoff, Vincent Hisler, Florian Baumgartner, Lana Rees, Dogan Grepper, Anna Jaźwińska

**Affiliations:** Department of Biology, University of Fribourg, Chemin du Musée 10, 1700 Fribourg, Switzerland

**Keywords:** Regeneration, Muscle stem cells

## Abstract

Skeletal muscles can regenerate after minor injuries, but severe structural damage often leads to fibrosis in mammals. Whether adult zebrafish possess the capacity to reproduce profoundly destroyed musculature remains unknown. Here, a new cryoinjury model revealed that several myomeres efficiently regenerated within one month after wounding the zebrafish caudal peduncle. Wound clearance involved accumulation of the selective autophagy receptor p62, an immune response and Collagen XII deposition. New muscle formation was associated with proliferation of Pax7 expressing muscle stem cells, which gave rise to MyoD1 positive myogenic precursors, followed by myofiber differentiation. Monitoring of slow and fast muscles revealed their coordinated replacement in the superficial and profound compartments of the myomere. However, the final boundary between the muscular components was imperfectly recapitulated, allowing myofibers of different identities to intermingle. The replacement of connective with sarcomeric tissues required TOR signaling, as rapamycin treatment impaired new muscle formation, leading to persistent fibrosis. The model of zebrafish myomere restoration may provide new medical perspectives for treatment of traumatic injuries.

## Introduction

Zebrafish have become leading model organisms for vertebrate tissue regeneration. Their complex organs, such as fins, the heart, and central nervous system structures, can be efficiently restored after major injuries by amputation, resection, or freezing^1–3^. Skeletal muscle regenerates after minor damage^4–8^. Whether extensive degeneration of locomotory musculature is followed by a restoration process has not yet been analyzed in adult zebrafish.

**Fig. 1:**
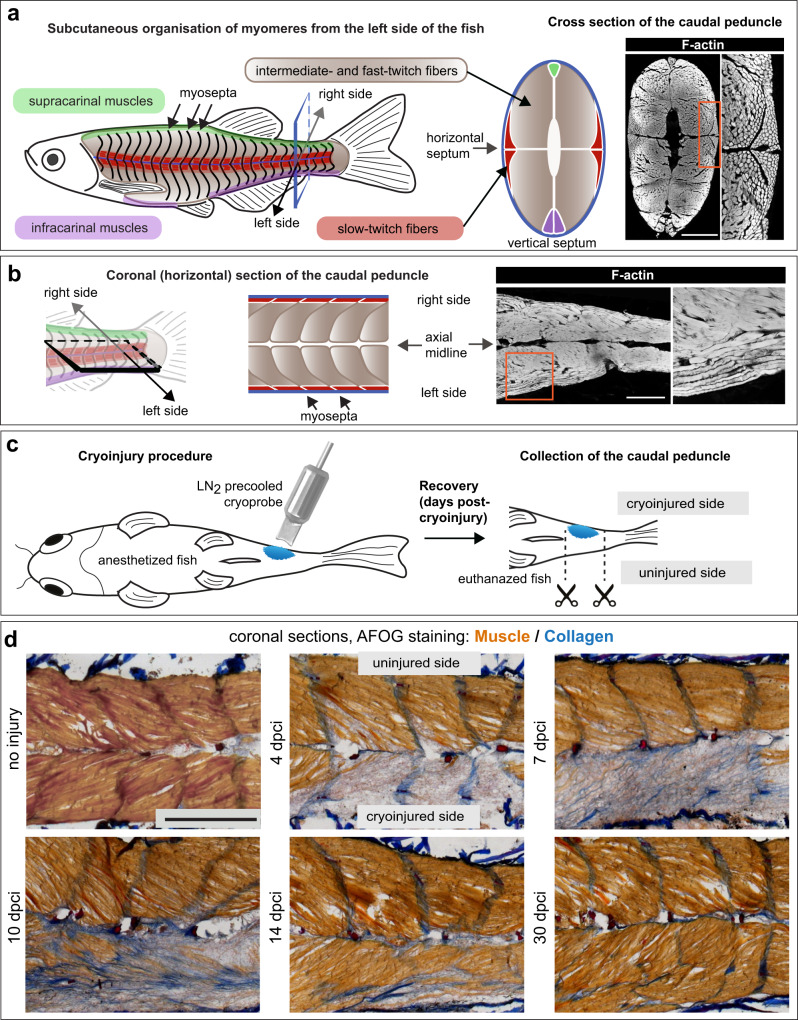
Myomere organization in adult zebrafish, and the effects of cryoinjury of the caudal peduncle. **a** Left side: Schematic of the musculature displays metameric organization of somite-derived units, called myomeres. Each myomere consists of superficial muscle (red area), which comprises slow-twitch fibers, and profound muscle (brown area), which contain fast- and intermediate-twitch fibers, grouped together as fast muscles. The upper- and lower-most margins comprise longitudinal muscles, called supracarinal muscles, along the dorsal side (green) and infracarinal along the ventral midline (purple). Right side: Transversal section through the caudal peduncle shown as a schematic and F-actin staining (white). The horizontal septum separates the dorsal and ventral parts. The vertical septum separates the left and right sides of the body. Scale bar indicates 500 μm. **b** Coronal section through the caudal peduncle shown as a schematic and F-actin staining (white). Scale bar indicates 500 μm. **c** Schematic of the cryoinjury procedure shown from the ventral side of the fish. The cryoprobe was precooled in liquid nitrogen (LN_2_) and immediately placed on one side of the anesthetized fish for 6 s. At a specific time point after cryoinjury, fish were euthanized, and their caudal peduncles were collected for fixation. **d** AFOG histological staining of coronal sections at different days post-cryoinjury (dpci). The uninjured side is detected by beige staining of the muscle, whereas the cryoinjured tissue lacks this staining. Collagen is stained in blue. At 4 and 7 dpci, three to four myomeres of one side appear grayish/bluish, corresponding to the wound area. At the subsequent points, new myofibers emerge at the injured side. Scale bar indicates 500 μm. *N* = 4.

The striated skeletal muscle is the most substantial tissue in the vertebrate body. In different fishes, skeletal muscles account for 40 to 70% of total weight^9–11^. The dorsal- and ventral-most margins of the body contain narrow longitudinal muscles, named supracarinal and infracarinal, which are poorly characterized^12,13^ (Fig. 1a). The lateral musculature is the most prominent, as it serves for side-to-side bending of the body to generate locomotory force. In contrast to mammals, fish maintain the segmental pattern of the somite-derived axial muscles throughout their lifespan. These metameric units, called myomeres or myotomes, correspond in number to the vertebrae, ranging between 20 to 120 in different species^11^. In zebrafish, 30 to 34 myomeres have been reported^14–16^. Sequential myomeres are interconnected by tissue sheaths, termed the myosepta or myocommata^15,17^. (Fig. 1a, b). Myomeres are superficially W-shaped. Transversally, each myomere is split into left and right sides, as well as dorsal and ventral parts, which are anchored by the midline septa. Thus, a myomere is a complex unit.

In the seminal work by Greene and Greene describing the musculature in salmon, the myomere is subdivided into the lateral profound muscle (in Latin *musculus lateralis profundus*), which forms the bulk of the musculature, and the lateral superficial muscle (*musculus lateralis superficialis*), lying in a relatively thin belt along both flanks of the body^12^. The superficial muscle consists of slow-twitch myofibrils, which have lower contractile force but prolonged activity that is sustained by aerobic metabolism. The profound muscle is composed of fast- and intermediate-twitch fibers, which are capable of burst activity, relying mostly on anaerobic (glycolytic) energetics. In our present study, both subtypes were integrated into the fast muscle group because distinctive molecular markers of their heterogeneity have not yet been sufficiently established. The physiological and developmental characteristics of these muscle types are considered to be similar to those of their mammalian counterparts^18–20^. The regenerative capacity of both muscle types after extensive myomere injury remains insufficiently characterized in adult zebrafish.

In mammals, muscle repair relies on tissue-specific stem cells, also called satellite cells^21,22^. These resident precursors are quiescent but can be stimulated to renew post-mitotic myofibers^23,24^. In zebrafish, lateral musculature also contains multinucleated myofibers and Pax7 expressing satellite-like cells, which are activated during regeneration^4,6,8,25–27^. Developmental studies are fundamental to understanding regeneration. Functional analysis revealed genes and cellular processes involved in myogenesis during zebrafish embryogenesis^27–34^. Much less is known about postembryonic muscle growth. As in mammals, muscle progenitors receive cues from their niche to undergo transition into myoblasts and finally mature myofibers^35^. The activation of satellite cells has been demonstrated after local chemical injury or stab wounding in larval and adult zebrafish^4–8^. However, in some other injury models, no evidence of stem cell activation has been identified. In zebrafish larvae, a severe disarray of somitic muscles after treatment with an acetylcholinesterase inhibitor could recover in a cell proliferation-independent manner^36^. In adult zebrafish, removal of 50% of the extraocular muscle is followed by regeneration with no evidence of satellite cells^37^. Furthermore, myonuclear reprogramming and dedifferentiation have been observed in this context^38–40^. In addition, fish can produce new myofibers in the process of eternal hyperplasia during ontogenic growth^41^. These studies suggest the existence of diverse regenerative mechanisms in teleosts. The lateral musculature with its myomeric organization might be particularly suitable to investigate the mechanisms of locomotory muscle regeneration after significant damage.

Here, we applied a cryoinjury procedure that has been developed in our laboratory to profoundly injure the musculature in one lateral side of the caudal peduncle. Targeting the posterior body, which lacks body cavities with viscera, decreases the risk of damage to inner organs. To monitor wound clearance and sarcomeric tissue restoration, we assessed markers of immune cells, autophagy, extracellular matrix, myogenesis and sarcomeres. To track the restoration of fast and slow muscles, we used transgenic reporter zebrafish lines and identified specific antibodies. To determine functional regeneration, cryoinjured fish were video recorded, and multiple swimming parameters were analyzed. At the molecular level, we show that muscle regeneration requires TOR signaling, as the inhibition of this pathway led to persistent fibrosis. This study demonstrates that complex myomeres are restored after severe injury, providing a new model system for studying the mechanisms of skeletal muscle regeneration in adult zebrafish.

## Results

### Collagenous matrix is transiently deposited during muscle restoration after cryoinjury

To damage a larger portion of locomotory muscles, we developed a cryoinjury procedure by applying a liquid nitrogen cooled metallic tool on the caudal peduncle^42^ (Fig. 1c). To determine the number of damaged myomeres, coronal sections were analyzed at several times after injury using AFOG histological staining, which labels the muscle in beige and collagen in blue (Fig. 1d). At 4 dpci (days post-cryoinjury), three to four consecutive myomeres of one flank were devoid of muscle staining, indicating the extent of damage. At 7 dpci, the wounded tissues contained scattered collagenous fibers, suggesting the formation of connective tissue. At 10 and 14 dpci, fibrotic tissue became infiltrated with a few muscle fibers. At 30 dpci, connective tissue was efficiently replaced with new muscle. At this stage, the regenerated zone could be traced by the misaligned myosepta, compared to the uninjured side of the body. In conclusion, cryoinjury depleted the muscle in several adjacent myomeres of one body side, but this loss was only transient, as new contractile tissue was re-established within one month.

**Fig. 2:**
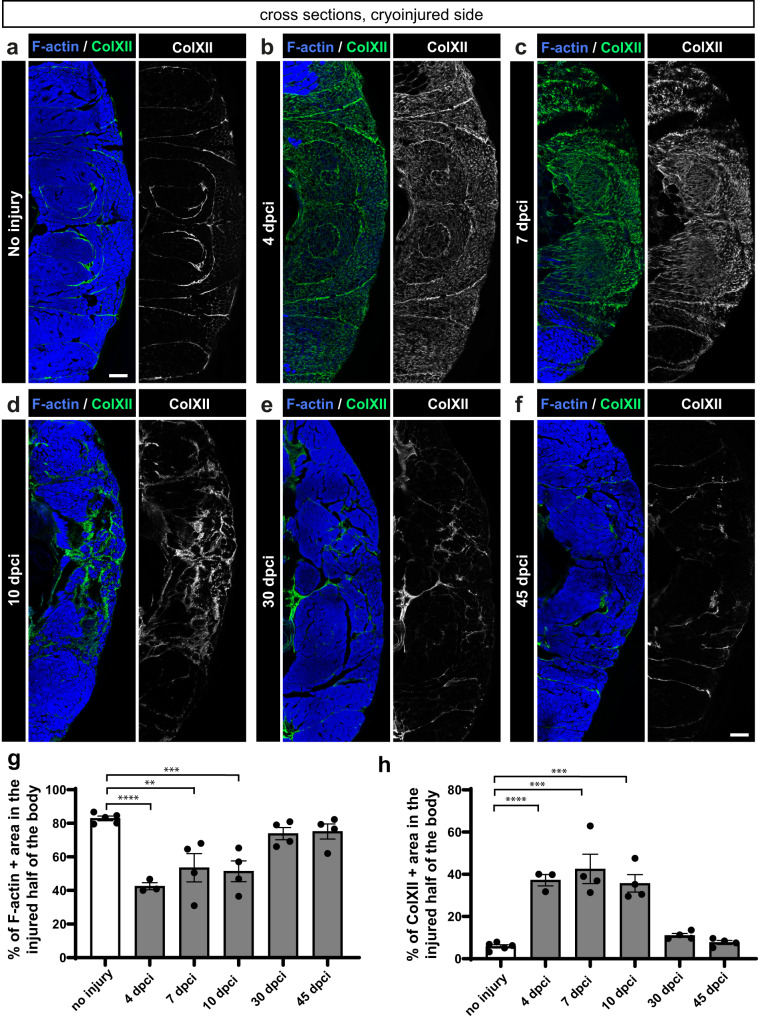
Dynamics of F-actin and Collagen XII in the wounded side of the fish body. **a**−**f** One half of cross sections fluorescently stained for ColXII and F-actin. In uninjured myomeres, ColXII demarcates the myosepta, which have a complex pattern in profound muscle due to the folded structure of the myomere^99^. At 4, 7 and 10 dpci, ColXII accumulates within the damaged area that is devoid of F-actin. At 30 and 45 dpci, new F-actin positive muscle replaced ColXII, which persisted in the myosepta, as in the original tissue. **g**, **h** Quantification of F-actin and ColXII was performed within the entire half of the body section (cryoinjured flank). *N* = 3 to 5 (one fish = one dot on the graph). One-way ANOVA with Tukey’s multiple comparisons test; error bar, SEM: (ns) not significant, (*) <0.05, (**) <0.01, (***) <0.001, (****) <0.0001. Skin and dermal scales emanating fluorescence outside the muscle area were erased from images using Adobe Photoshop for clarity in this and the subsequent figures. Scale bar indicates 100 μm.

The AFOG staining revealed a complementary pattern of muscle and collagenous tissue in the damaged flank. To quantify the dynamics between both tissues, we performed fluorescence staining against F-actin, as a myofiber marker, and Collagen XII (ColXII), a nonfibrillar collagen that is upregulated during heart and spinal cord regeneration in zebrafish^43,44^. For the assessment of entire myomeres, cross sections were performed and analyzed. Firstly, the amount of muscle per section area at several time points was measured. In uninjured samples, F-actin-positive area occupied approximately 80% of the body side (Fig. 2a, g). At 4 dpci, F-actin area decreased to approximately 40% of the injured side (Fig. 2b, g). A mild restoration of muscle up to approximately 50% was detected at 7 and 10 dpci (Fig. 2c, d, g). At 30 and 45 dpci, F-actin positive area was similar to control, consistent with muscle restoration (Fig. 2e−g).

Next, we assessed ColXII staining of the same specimens. In uninjured muscle, ColXII was localized at the myosepta, which occupied approximately 6% of the sectioned area (Fig. 2a, h). At 4, 7 and 10 dpci, the wound area displayed a dense mesh-like network of ColXII within approximately 40% of the injured side (Fig. 2b−d, h). At 30 and 45 dpci, ColXII outlined the myosepta of regenerated myomeres, returning to the nearly original pattern (Fig. 2e, f, h). These findings demonstrate that extensive deposition of ColXII was transient during the initial phase, and it resolved with progressing regeneration. To conclude, myomere regeneration does not involve a permanent scar.

### Degeneration of muscle is accompanied by cell infiltration and an immune response

**Fig. 3:**
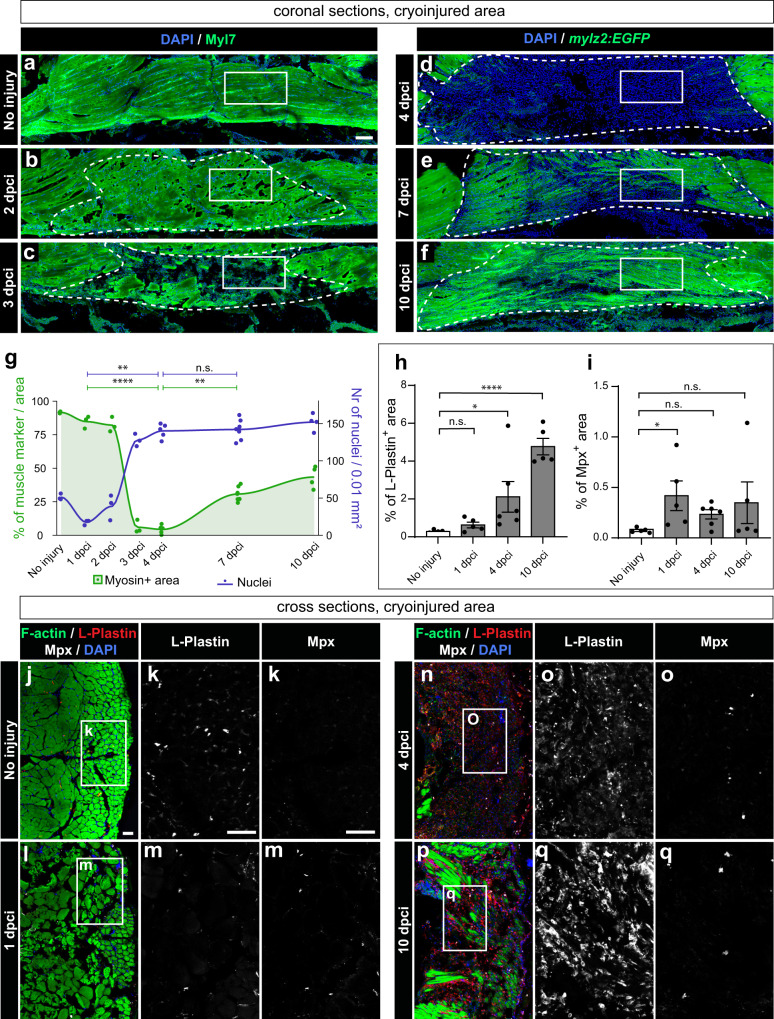
Muscle clearance is associated with cell infiltration and an immune response. **a**−**f** Coronal sections fluorescently stained for four markers, of which the figure displays muscle staining (green) and nuclei (DAPI, blue), whereas two other markers (Pax7 and PCNA) are shown in (Supplementary Fig. 2 and Fig. 4). In panels (**a**, **b**, **c**), wild-type fish were used, and the muscle marker was MYL7 antibody, whereas in panels (**d**, **e**, **f**), *mylz2:EGFP* fish were used, and the profound muscle was detected with the GFP antibody. White frames demarcate the areas that are shown in (Fig. 4), displaying PCNA, Pax7 and DAPI. Cryoinjured areas are encircled with a dashed line, drawn based on the distorted staining of the muscle marker. For uninjured fish, a similar area was considered. **g** Quantification of muscle marker within the encircled cryoinjured area (left y-axis: muscle+ area, green) and cell number (right y-axis: DAPI+ nuclei, dark blue). *N* = 3 to 7 fish per time-point (one fish = one dot, an average of several sections per fish). Dunn’s test: (ns) not significant, (*) <0.05, (**) <0.01, (***) <0.001, (****) < 0.0001. The muscle underwent resorption at 3 and 4 dpci, which correlated with a significant increase in the number of cells within the wound area. Scale bar indicates 100 μm. Quantification of immune cell markers, L-Plastin (**h**) and Mpx (**i**) in cross sections that representatively shown below. The marker area is calculated as percentage of the cryoinjured side of the body. The measurements were performed within the entire half of the body section (cryoinjured side). *N* = 5 or 6 fish per time-point. Error bar = SEM. One-way ANOVA with Tukey’s multiple comparisons test. The meaning of the stars is the same as in (**g**). **j**−**q** Representative images of uninjured control or cryoinjured side, immunostained for L-plastin and Mpx, counterstained with phalloidin (F-actin) and DAPI. The frame indicates the area that is magnified in the images to the right. Scale bar indicates 50 μm.

To gain insights into the dynamics of muscle clearance within the damaged tissue, coronal (horizontal) sections were analyzed using a profound muscle marker and nuclear staining. At 1 and 2 dpci, the amount of Myosin light chain 7 (Myl7) in the injured area was not substantially different than in control (Fig. 3a, b, g). A rapid loss of muscle in the wounded zone was observed at 3 dpci (Fig. 3c, g). The next time points were analyzed using the transgenic fish *mylz2:EGFP*, which demarcates the profound muscle^45^. At 4 dpci, the wounded area was devoid of muscle remnants, suggesting an efficient resorption of the damaged tissue (Fig. 3d, g). Interestingly, muscle degradation correlated with a rapid increase in DAPI stained nuclei, suggesting a massive infiltration and/or proliferation of cells in the damaged zone. At 7 and 10 dpci, the amount of muscle started to increase (Fig. 3e, f, g). In conclusion, the degeneration of sarcomeric tissue was associated with cell infiltration at 3 and 4 dpci.

The breakdown of cellular components might involve cell survival pathways triggered by cryoinjury^46^. To assess whether the autophagic response is stimulated during muscle degradation, we performed immunofluorescence staining for p62, also called Sequestosome 1 (SQSTM1). This protein acts as a receptor for capturing ubiquitinated cargo, such as macromolecules or organelles, delivering them to the autophagosomes^47–49^. Analysis of cross sections at 1 and 3 dpci revealed that damaged F-actin positive myofibers contained a punctate pattern of p62 staining, consistent with vesicular subcellular localization (Supplementary Fig. 1). Such p62 immunoreactivity was not detected in the respective area of the uninjured flank of the body, which contained regular myofibrils. The presence of p62 puncta on the cryoinjured side suggests the involvement of autophagy during tissue clearance.

An increase in nuclear staining during muscle degeneration might be associated with recruitment of immune cells. To assess the distribution of phagocytes (mainly macrophages and some neutrophiles), we performed immunostaining against L-plastin, a leukocyte-specific actin-bundling protein^50,51^. To demarcate neutrophils, immunostaining against myeloperoxidase (Mpx, also abbreviated as Mpo) was applied^52^. The number of these immune cells was quantified in cross sections. In comparison to uninjured control, L-plastin positive cells increased by 2-, 7- and 17-fold at 1 dpci, 4 dpci, and 10 dpci, respectively, suggesting a progressive enrichment of phagocytes at the injury zone (Fig. 3h−q). Mpx-positive neutrophils slightly increased at 1 dpci, but at the next time-points, they returned to similar levels as in control. These data suggest that the immune response involves a gradual recruitment of L-plastin expressing phagocytes during the first 10 days after injury.

### Loss of skeletal muscle activates the Pax7- and MyoD1-dependent myogenic program

**Fig. 4:**
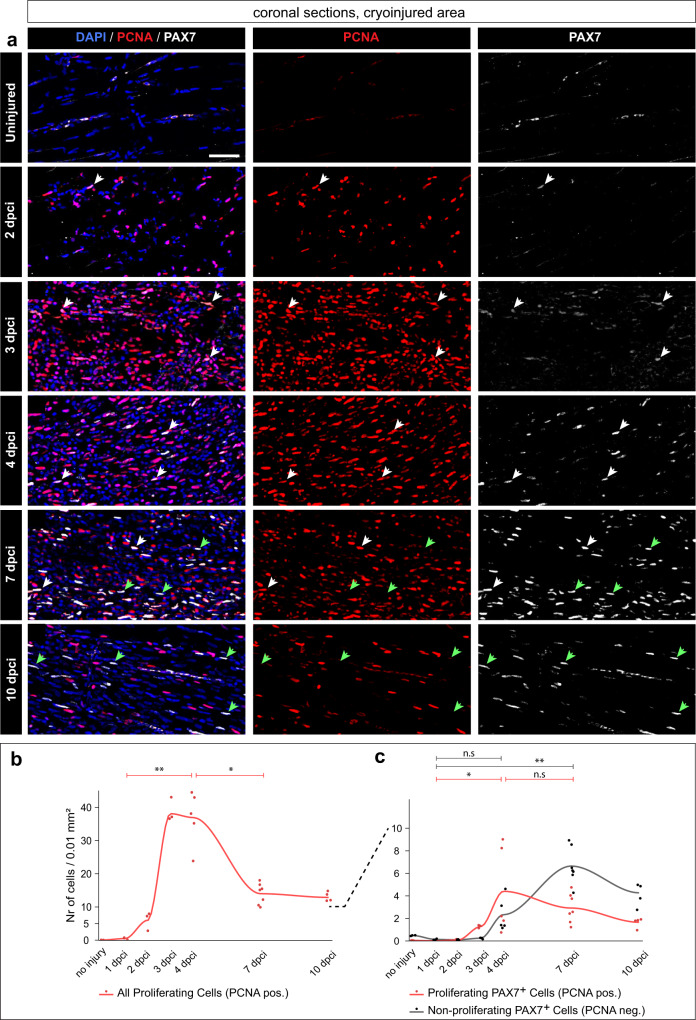
An increase of PAX7+ satellite cells and cell proliferation in the wound after muscle degeneration. **a** Coronal sections fluorescently stained for four markers, of which the figure displays Pax7 (gray), PCNA (red) and DAPI (blue). The non-displayed channel is muscle staining shown in (Fig. 3a−f). The images are enlargements of the framed areas shown in (Fig. 3a−f and Supplementary Fig. 2, in which the encircled areas demarcate the region used for quantification). White arrows depict proliferating muscle stem cells (PCNA + PAX7 + DAPI+ nuclei); green arrows indicate non-proliferating muscle stem cells (PCNA negative PAX7 + DAPI+ nuclei). Scale bar indicates 50 μm. **b**, **c** Quantification of Pax7 and PCNA nuclear staining per 0.01 mm^2^ area of the cryoinjured area or its respective control in uninjured fish (encircled area in Supplementary Fig. 2). *N* = 3 to 7 fish per time-point (one fish = one dot, an average of several sections per fish). Dunn’s test: (ns) not significant, (*) <0.05, (**) <0.01, (***) <0.001, (****) <0.0001. **b** Dynamics of all proliferating cells (PCNA + DAPI+ nuclei, red). **c** Dynamics of proliferating muscle stem cells and non-proliferating muscle stem cells, as specified above.

To determine the dynamics of muscle stem cells and their proliferation during the onset of regeneration, we assessed Paired box 7 (Pax7) and PCNA. Immunodetection for these markers was performed as a quadruple staining together with a muscle marker and DAPI on coronal sections (Fig. 3a−f and Supplementary Fig. 2). Pax7 and PCNA positive cells colocalized with DAPI, and they were quantified in the injured area, as demarcated by abnormal/deficient muscle staining. Firstly, we focused on all proliferating cells. PCNA positive cells were nearly undetectable in uninjured fish and at 1 dpci, however, they were strongly detected at 3 and 4 dpci (Fig. 4a, b). Their number was reduced by half at 7 and 10 dpci (Fig. 4a, b). This suggests that the burst of cell proliferation coincided with muscle degradation and cell infiltration, as shown above (Fig. 3a−f).

Next, we focused on proliferating and non-proliferating muscle stem cells. In uninjured control, at 1 and 2 dpci, we detected very few Pax7 positive cells (Fig. 4a, c). At 3 dpci, the injured zone showed a higher number of Pax7 cells, among which nearly all were also PCNA positive (Fig. 4a, c). At 4 dpci, PCNA/Pax7 double positive cells were highly abundant in the wound and this number mildly decreased at 7 and 10 dpci (Fig. 4a, c). This suggests that during muscle degradation, muscle stem cells rapidly enter the cell cycle, and then they progressively return to quiescence during the formation of new fibers.

MyoD1 (Myogenic differentiation or Myoblast determination 1) is a transcription factor of muscle progenitors^29^. Double staining of Pax7 and MyoD1 revealed the presence of double positive nuclei in the regenerating tissue (Supplementary Fig. 3a). This observation suggests that muscle regeneration involves a transition from muscle stem cells to committed myoblasts. Surprisingly, beside the nuclear localization of MyoD1, we observed a cytoplasmic immunoreactivity of the MyoD1 antibody. To further understand this rather unexpected finding, we analyzed uninjured specimens in combination with two monoclonal antibodies from Developmental Study Hybridoma Bank (DSHB), recognizing Troponin T2 (Tnnt2)^53^, and N2.261 for an isoform of Myh7 and ß-cardiac myosin^54,55^. In combination with F-actin labeling, triple immunostaining revealed colocalization between MyoD1, Tnnt2 and Myh7 in the superficial muscle (Supplementary Fig. 3b). Using technical controls, we determined that MyoD1 cytoplasmic staining was not an imaging artifact (Supplementary Fig. 3c).

**Fig. 5:**
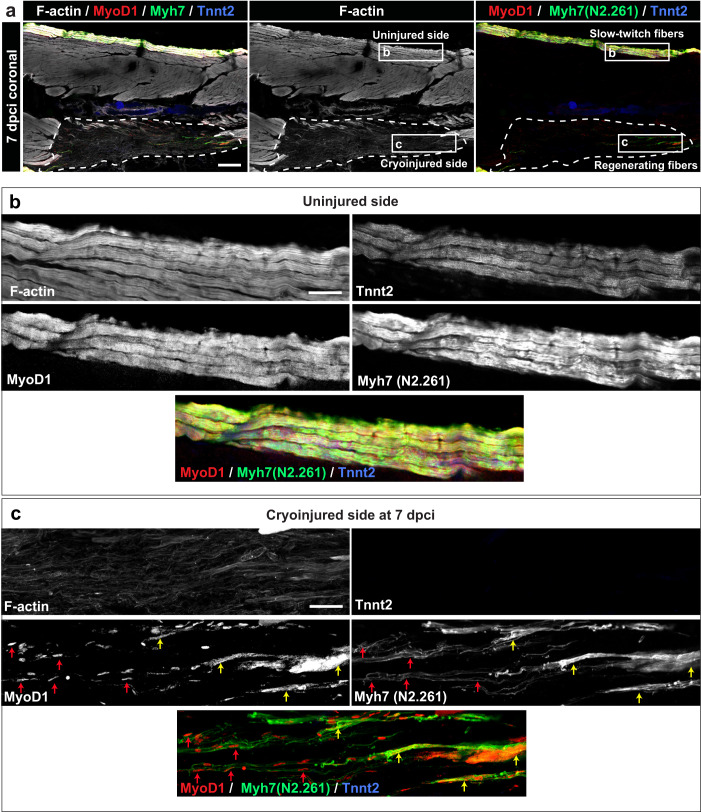
Expression of MyoD1 and slow muscle markers in early regenerating myofibers. **a** Quadruple fluorescence staining of a coronal section at 7 dpci. A dashed line encircles the wound. The frames depict the areas that are enlarged in panels below. *N* = 3 fish. Scale bar indicates 200 μm. **b** A higher magnification of the uninjured side in the framed area as indicated in (**a**). The superficial fibers co-express F-actin, Tnnt2 (CT3 antibody), MyoD1 an Myh7 (N2.261 antibody). Scale bar indicates 50 μm. **c** A higher magnification of the cryoinjured side in the framed area as indicated in (**a**). The wounded area contains scattered myogenic precursors that are demarcated by nuclear MyoD1 immunoreactivity (red arrows), associated with fine Myh7 fibrils, which seem to outline an elongated morphology of cells. Near the wound margin (right side of the images), myogenic cells display cytoplasmic MyoD1 immunoreactivity and thicker Myh7 fibrils, both of which are partially overlapping (yellow arrows). Little F-actin and no Tnnt2 was detected in the wound, indicating the absence of sarcomeric tissue. Scale bar indicates 50 μm.

To assess the expression of these muscle markers during new myofiber formation, we analyzed specimens at 7 dpci. In the uninjured side, as shown above, MyoD1, Tnnt2 and Myh7 colocalized in the superficial muscle (Fig. 5a, b). In the cryoinjured side, MyoD1 staining displayed two subcellular patterns in the tissue with low F-actin and no Tnnt2 immunoreactivity. Firstly, nuclear MyoD1 was associated with thin Myh7 positive fibrils, which seemed to outline the shape of new myogenic cells in the wound (Fig. 5c, red arrows). Secondly, cytoplasmic MyoD1 colocalized with thicker Myh7 positive fibrils, which were localized at the wound periphery (Fig. 5c, yellow arrows). The latter cells might represent a more advanced myogenic state than the former ones. Taken together, during the first week post-cryoinjury, the myogenesis program proceeds from proliferation of Pax7 positive satellite-like cells, a transition into MyoD1 positive myocytes, followed by formation of immature myofibers that express the N2.261-immunoreactive Myh7 isoform. The undifferentiated stage of the muscle was defined by markedly lower F-actin, compared to mature muscle.

### Early myofibers display a slow-twitch muscle-like identity

Slow muscles have been previously demarcated using the F59 antibody^14,56^. We found that F59 immunostaining was relatively weak, nevertheless it colocalized with Tnnt2 detected with the CT3 antibody (Supplementary Fig. 4). These results confirm the slow muscle identity of Tnnt2-expressing myofibers.

**Fig. 6:**
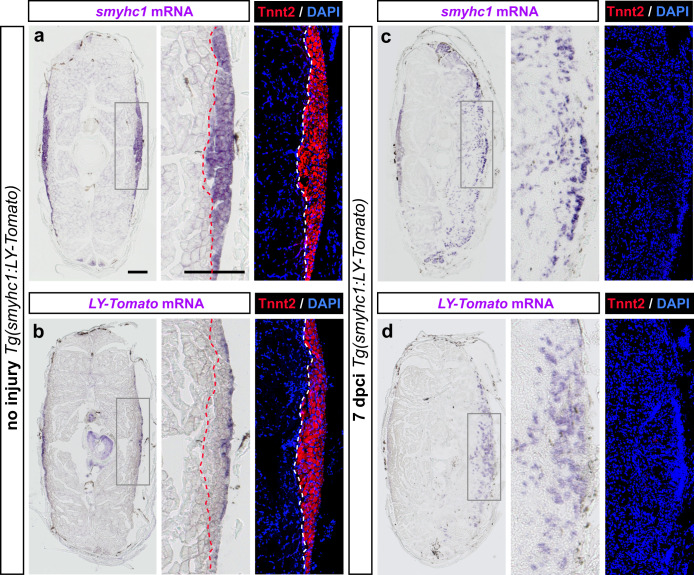
Comparison between *smyhc1* and *smyhc1:LY-Tomato* expression in uninjured and regenerating muscles. Transversal sections of *smyhc1:LY-Tomato* transgenic fish, stained by in-situ hybridization (purple), Tnnt2 antibody (red) and DAPI (blue). *N* = 3 fish per group. The frames depict the areas that are enlarged in the images to the right. Scale bar indicates 200 μm. **a**, **b** In uninjured fish, Tnnt2 positive muscle is outlined with a dashed line. The *smyhc1* probe (**a**) labels the entire Tnnt2 positive area, corresponding to the slow muscle. The *LY-Tomato* probe (**b**) detects only the superficial-most layer of the slow muscle. **c**, **d** At 7 dpci, the *smyhc1* probe (**c**) displays a scattered labeling in the cryoinjured side, but no Tnnt2 staining. A similar expression is detected for the *LY-Tomato* probe (**d**). Scale bar indicates 200 μm.

The slow muscle type can be further identified by the expression of *slow muscle myosin heavy chain 1* (*smyhc1*) gene^57,58^. As we aimed to use a fluorescent reporter of this gene, *smyhc1:LY-Tomato*, we first conduced in-situ hybridization with *smyhc1* and *LY-tomato* antisense probes to compare their localization on cross sections. In uninjured fish, *smyhc*1 transcripts were detected in several layers of the superficial muscle that entirely colocalized with Tnnt2 immunostaining (Fig. 6a). By contrast, the *LY-tomato* probe was restricted to one or two superficial layers of myofibers in a subset of Tnnt2 expressing cells (Fig. 6b). This suggests that *smyhc1:LY-Tomato* demarcates only a superficial-most layer of the slow muscle compartment.

To determine whether the *smyhc1* reporter is suitable for studying myofiber reappearance, we analyzed the expression of the *smyhc1* gene and the *tomato* transgene after cryoinjury. At 7 dpci, both *smyhc1* and *LY-tomato* antisense probes detected scattered fibers in the injured side (Fig. 6c, d). Tnnt2 was not expressed at this time point, consistent with our previous findings. Thus, the new immature myofibers seem to transiently activate *smyhc1* expression across the wound depth.

To investigate whether the *smyhc1* reporter marked newly formed myofibers, the expression of *smyhc1:LY-Tomato* and MyoD1 were analyzed on coronal sections at the same time-point. A positional correlation between *smyhc1:LY-Tomato*-positive tissue and MyoD1-positive nuclei was observed in regenerating myofibers (Supplementary Fig. 5a). We further tested whether the *smyhc1:LY-Tomato* expression was not an artefact of the specific transgene. To this aim, *smyhc1:LY-Tomato* fish were crossed with *smyhc1:EGFP* fish. At 7 dpci, *smyhc1:LY-Tomato; smyhc1:EGFP* double transgenic fish displayed a similar pattern of both fluorescent proteins in the wounded area (Supplementary Fig. 5b). This validates the specificity of the observed pattern for both *smyhc1* reporters. Taken together, these findings suggest that the *smyhc1* regulatory element serves as a reporter of regenerating myofibers and that early myofibers seem to have a slow twitch-like identity.

### Monitoring of slow and fast muscle regeneration with transgenic reporters

**Fig. 7:**
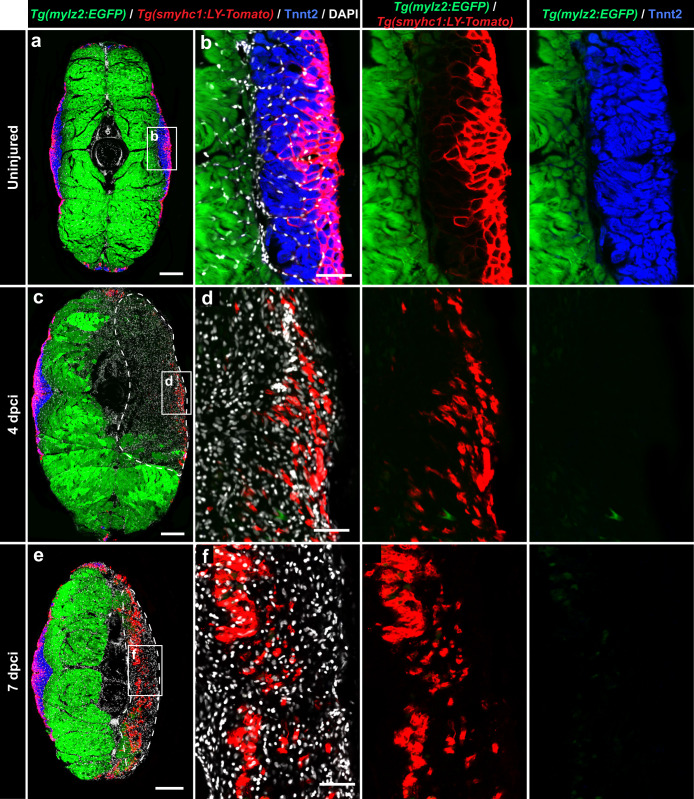
The *smyhc1:LY-Tomato* transgenic reporter is upregulated in regenerating myofibrils. **a**−**f** Cross sections of the *mylz2:EGFP* and *smyhc1:LY-Tomato* double transgenic fish demarcating fast-twitch myofibers of the profound muscle (endogenous EGFP, cytosolic localization, green) and superficial layers (anti-Tomato/Cherry immunostaining, plasma membrane localization, red) of the slow muscle compartment, which is immunolabeled with Tnnt2 antibody (blue). All nuclei are stained with DAPI (white). *N* = 3 (control) to 5 fish (test groups), several sections per fish were analyzed. Scale bar in (**a**, **c** and **e**) indicates 200 μm. Scale bar in (**b**, **d** and **f**) indicates 50 μm. **a**, **b** In uninjured samples, a complementarity is observed between the fast muscle marker (*mylz2:EGFP*) and a slow muscle marker, Tnnt2. LY-Tomato immunoreactivity outlines myofibers in the outer layers of the Tnnt2-positive compartment. **c**, **d** At 4 dpci, the cryoinjured area is devoid of fast muscle (*mylz2:EGFP*) and slow muscle (Tnnt2) markers. In the outer layers, LY-Tomato immunoreactivity is scattered in the wound. **e**, **f** At 7 dpci, LY-Tomato immunoreactivity appears in the inner region of the damaged flank, suggesting the activation of *smyhc1:LY-Tomato* expression in new regenerating myofibers.

To assess dynamics of slow and fast muscle regeneration, we used *mylz2:EGFP* and *smyhc1:LY-Tomato* double transgenic fish, demarcating fast- and superficial slow-twitch muscles, respectively^45,59–62^. For detection of LY-Tomato, which is a membrane-anchored protein, immunostaining against the fluorescent reporter was necessary, whereas the Tnnt2 antibody was applied to identify all slow muscles. In uninjured fish, Tnnt2 and *mylz2:EGFP* positive tissues were nonoverlapping and complementary to each other, consistent with the expected spatial separation of slow- and fast-twitch muscles (Fig. 7a, b, Supplementary Fig. 6h). As showed before, *smyhc1:LY-Tomato* labeled the subcutaneous region of the Tnnt2-positive tissue (Fig. 7a, b, Supplementary Fig. 6g).

The wound area was identified by the interrupted expression of *mylz2:EGFP* and depleted Tnnt2 expression (Fig. 7c−f). After cryoinjury, this double transgenic fish revealed a clearance of muscles (Supplementary Fig. 6a), correlating with a shrinkage of the damaged tissue (Supplementary Fig. 6b) and an increase in cell number (Supplementary Fig. 6c), which are consistent with the previous results (Fig. 3a−g).

To determine the change in fast and slow muscles after cryoinjury, we quantified the expression of the muscle markers within the wound and its respective mirror-imaged area of the control side. At 4 and 7 dpci, *mylz2:EGFP* was nearly absent in the wound, indicating damage to fast-twitch muscle fibers (Fig. 7d, f, Supplementary Fig. 6d). Similarly, Tnnt2 expression was not detected in the cryoinjured area, suggesting a loss of slow muscles (Fig. 7d, f, Supplementary Fig. 6f). *smyhc1:LY-Tomato* appeared at 7 dpci in the inner zone of myomeres (Fig. 7f, Supplementary Fig. 6e), consistent with in-situ hybridization data. Given that *smyhc1:LY-Tomato* expression correlated with decreased proliferation of Pax7-positive cells (Fig. 4c), the appearance of MyoD1-positive nuclei (Supplementary Fig. 5a) and myosin expression (Figs. 3g, 5c), we concluded that this transgene demarcates the entry into the differentiation stage of newly formed myofibers, irrespectively of their final identity.

### Complete restoration of slow and fast muscles after cryoinjury

**Fig. 8:**
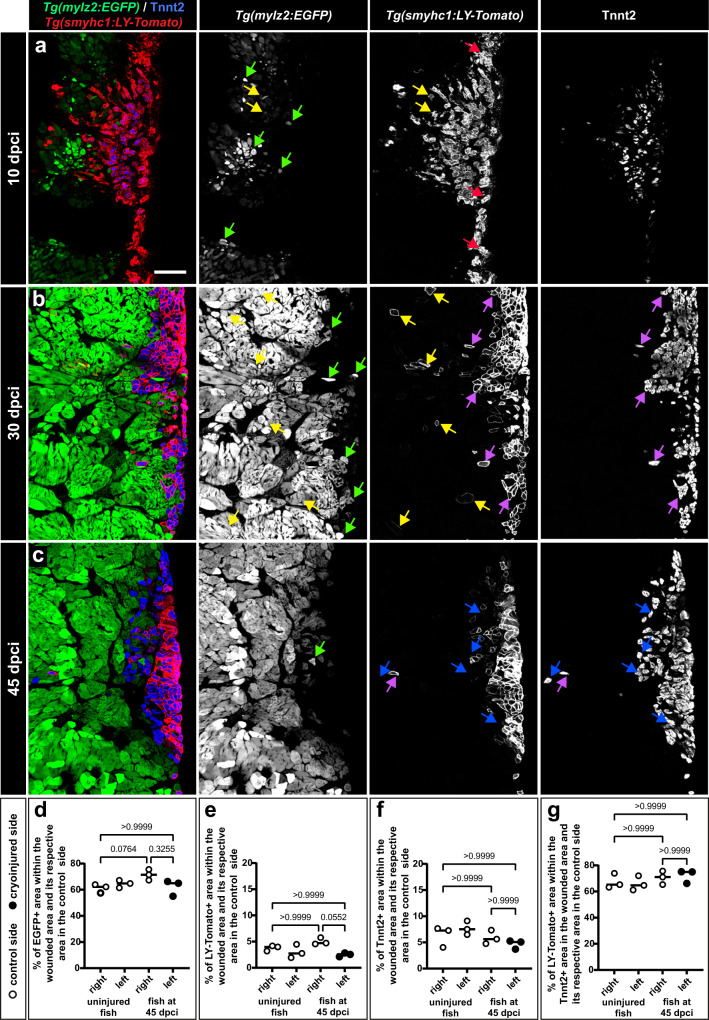
A nearly perfect restoration of fast and slow myofibers is accomplished at 30 to 45 dpci. Higher magnifications of cross sections displaying the injured side of *mylz2:EGFP* and *smyhc1:LY-Tomato* double transgenic fish. Sections were immunostained for Tnnt2 (slow muscles) and for Tomato. Scale bar indicates 100 μm. **a** At 10 dpci, all three markers are activated in the wounded area, suggesting advancing regeneration. Most *mylz2:EGFP* positive fibers (fast myofibers) are negative for LY-Tomato and Tnnt2 (green arrows). The plasma membrane of superficial muscles is immunoreactive for the Tomato antibody (*smyhc1:LY-Tomato*; red arrows). Some of these cells also express Tnnt2. A few LY-Tomato positive cells are also positive for *mylz2:EGFP* (yellow arrows). **b** At 30 dpci, the wounded tissue is filled with muscle fibers. A few *mylz2:EGFP* single positive myofibers spread into the superficial muscle (green arrows). Some *mylz2:EGFP* positive myofibers also express *smyhc1:LY-Tomato* (yellow arrows). Most Tnnt2-positive cells are also Tomato positive cells (purple arrows). **c** At 45 dpci, nearly no overlap between *mylz2:EGFP* and *smyhc1:LY-Tomato* expression suggests terminal differentiation of fast and slow muscle identities. Most of *mylz2:EGFP* positive fibers retract from the superficial layers, and only a very few individual cells intermingle in the slow muscle compartment (green arrow). Tnnt2 positive cells (blue arrows) form a wedge-like compartment, whereas Tnnt2/LY-Tomato accumulate at the superficial layers, thus, both of which return to the original pattern. Only individual cells that are Tnnt2 or/and LY-Tomato positive remain mislocalized in the fast muscle compartment. Thus, the normal strict separation between fast and slow muscle fibers is still not perfectly re-established. **d**−**g** Quantification of fluorescent areas within the wounded area (left side) compared to its respective area of the control side (right side), as indicated on the graphs. White points, uninjured side; black points, cryoinjured side. *N* = 3 fish, each representing an average of 3 nonadjacent sections. Error bar, SEM. Kruskal-Wallis test with Dunn’s post hoc analysis.

To determine differentiation of fast and slow muscles, we continued analysis of *smyhc1:LY-Tomato; mylz2:EGFP* double transgenic fish at later time-points. At 10 dpci, *mylz2:EGFP* and Tnnt2 expression, which were not observed in the cryoinjured area at 7 dpci, started to emerge in the regenerating muscle (Fig. 8a). Remarkably, the intensity of fluorescent labeling was very heterogenous, suggesting an ongoing transition from myogenic precursors to terminal differentiation. Furthermore, some of the *mylz2:EGFP* fibers were also positive for *smyhc1:LY-Tomato*, consistent with the finding that the *smyhc1* reporter demarcates an early myofiber state before terminal differentiation (Fig. 8a, yellow arrows). No co-expression was found between *mylz2:EGFP* and Tnnt2, suggesting a strict separation between fast- and slow-twitch myofibers.

At 30 dpci, *mylz2:EGFP* positive fibers filled out the wound, demonstrating the restoration of fast-twitch muscles (Fig. 8b). Some myofibers were *smyhc1:LY-Tomato* and *mylz2:EGFP* positive, corresponding to an incomplete differentiation state of fast muscle (Fig. 8b, yellow arrows). Other myofibers were *smyhc1:LY-Tomato* and Tnnt2 double positive, representing a differentiating slow muscle (Fig. 8b, purple arrows). Unlike in the original pattern, scattered *mylz2:EGFP* single positive myofibers were intermingling in the superficial muscle (Fig. 8b, green arrows). At 45 dpci, *mylz2:EGFP* positive fibers retracted from the subcutaneous position, demarcating a partial recovery toward the separation of fast and slow myofibers within their specific compartments (Fig. 8c). Occasionally, a few individual slow myofibers were mislocalized in the profound muscle (Fig. 8c). Beside these exceptions, *smyhc1:LY-Tomato* expression was restricted to the subcutaneous subregion of the Tnnt2 positive tissue, returning to the original pattern.

To quantify the regeneration level, we compared the fluorescent areas between the cryoinjured (left) and uninjured (right) sides. All markers displayed similar values between the control and cryoinjured sides of the body (Fig. 8d−g, right and left sides). Comparison between uninjured fish and those at 45 dpci also revealed no difference. These findings demonstrate the full restoration of slow- and fast-twitch muscle fibers after cryoinjury.

The presence of some mislocalized fast and slow myofibers suggests imperfect architecture of the regenerated musculature. To exclude the possibility that this phenotype was associated with the transgenic background, we analyzed wild-type fish using F-actin and Tnnt2 staining. At 45 dpci, the uninjured control side displayed a smoothly curved boundary between slow- and fast-twitch muscles, as expected, whereas the regenerated boundary acquired a zigzag-like appearance (Supplementary Fig. 7a−d). Thus, the spatial compartmentalization between different myofiber types was less stringent in the regrown skeletal muscle than in the original pattern. The quantification of F-actin and Tnnt2 demonstrated nearly complete reproduction of the muscle at 45 dpci (Supplementary Fig. 7e−g). These parameters support the conclusion of efficient skeletal muscle regeneration after myomere cryoinjury, although the slow/fast muscle boundary lost its original smooth shape.

**Fig. 9:**
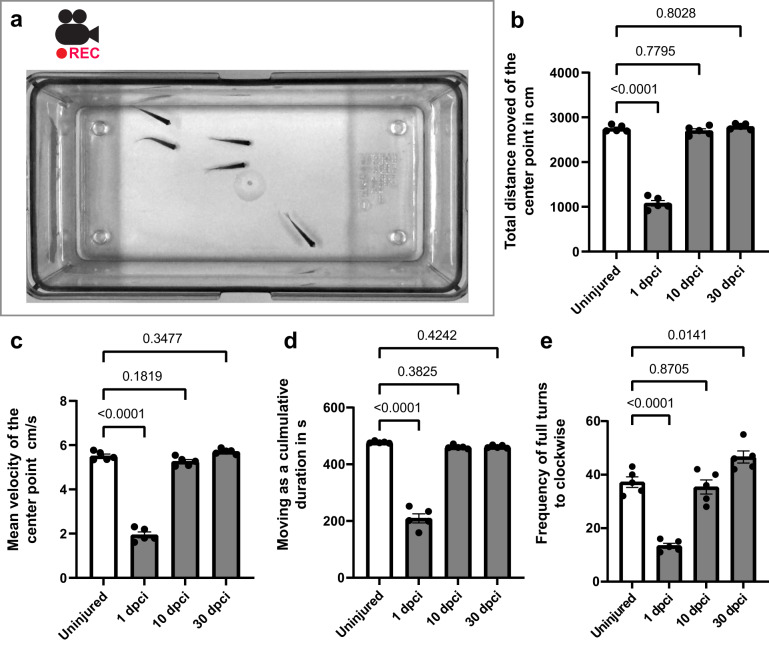
Zebrafish retain swimming activity after cryoinjury. **a** A snapshot of the movie. After one minute of acclimatization in a new tank, a group of 5 fish was filmed for ten minutes. **b**−**e** Statistical analysis of swimming parameters, as indicated on graphs at 1, 10 and 30 dpci compared to controls using ten-minute videos. Error bar, SEM. One-way ANOVA with Dunnett’s post hoc analysis.

To determine whether swimming performance was affected after cryoinjury, a 10 min video recording of fish was performed (Fig. 9a, Supplementary Movies 1−3). At 1 dpci, several mobility parameters were approximately twice as low as those in the uninjured control, namely, total swimming distance, mean velocity, cumulative duration, and frequency of turns (Fig. 9b−e). These values suggest that the cryoinjured fish were swimming less actively. Nevertheless, the fish did not display any abnormal movements, such as swirling, convolution or reduced equilibrium. Their position in the tank and feeding behavior were normal. At 10 and 30 dpci, all swimming parameters were similar between cryoinjured and uninjured fish, except frequency of turns, which was slightly higher at 30 dpci than in uninjured fish. This behavioral change might be due to some asymmetry between the uninjured and regenerated muscles at the right and left sides of the body. To conclude, cryoinjury of the caudal peduncle only mildly and transiently disturbed the locomotion of the fish at standard conditions.

### Inhibition of TOR signaling impairs muscle regeneration

**Fig. 10:**
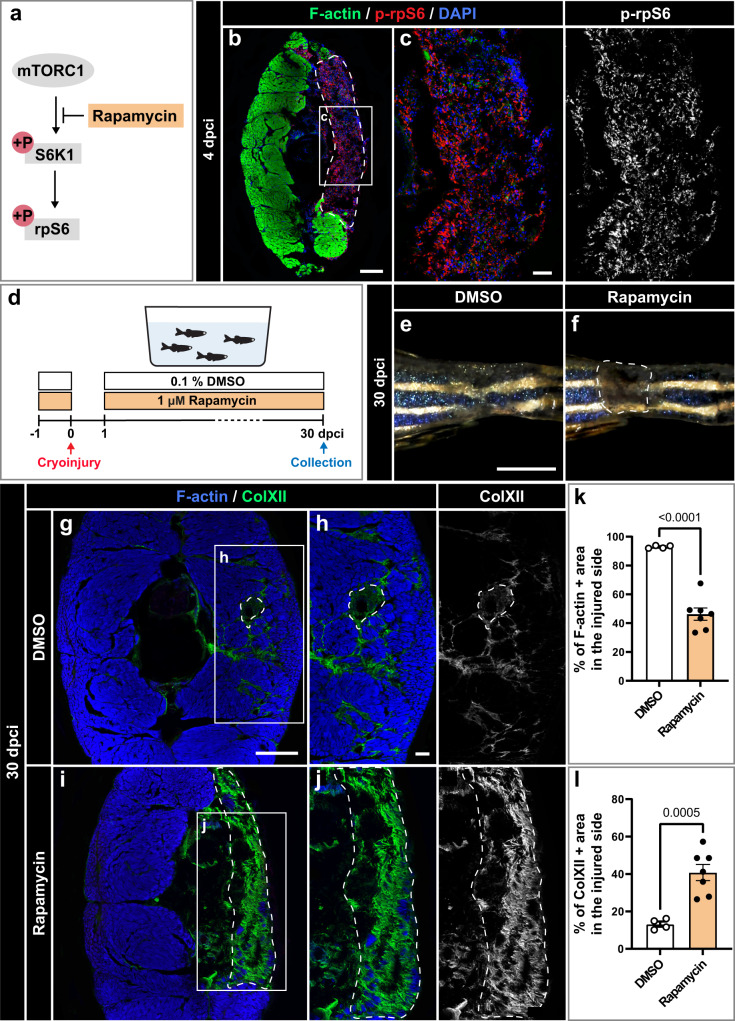
TOR signaling is required for muscle regeneration. **a** Schematic illustration of mammalian TORC1 signaling with the indication of its inhibitor rapamycin. Phosphorylation of ribosomal protein S6 (p-rpS6) provides a readout for the pathway activity. **b**, **c** At 4 dpci, cross sections display p-rpS6 immunoreactivity in the wounded area. *N* = 3. Scale bar in (**b**) indicates 200 μm. Scale bar in (**c**) indicates 50 μm. **d** Experimental design with 1 µM rapamycin or 0.1% DMSO, which is the control condition. **e**, **f** Photos of the caudal peduncle. The persisting wound is encircled with a dashed line in (**f**). Scale bar in (**e**) indicates 2 mm. **g**−**j** At 30 dpci, cross sections were stained for F-actin and ColXII. DMSO-treated control fish demonstrated advanced regeneration, whereas rapamycin-treated fish displayed extensive fibrotic tissue in the wound. Scale bar in (**g**) indicates 200 μm. Scale bar in (**h**) indicates 50 μm. Quantification of F-actin (**k**) and ColXII (**l**) at 30 dpci. Quantification of F-actin and ColXII was performed within the entire half of the body section (the cryoinjured lateral side). DMSO group: *N* = 4; rapamycin group: *N* = 7. Error bar, SEM. Unpaired two-tailed Student’s *t* test.

One of the common regulators of tissue regeneration is the kinase activity of mammalian Target of Rapamycin (TOR)^63,64^. To determine whether this pathway is involved in skeletal muscle regeneration after cryoinjury, we applied an antibody against phosphorylated ribosomal protein S6 (p-rpS6), which is a readout of mTORC1-dependent signaling in mammals and zebrafish^65^ (Fig. 10a). At 4 dpci, p-rpS6 immunoreactivity was abundantly detected in the cryoinjury zone of DMSO-treated control fish, suggesting the activation of the TOR pathway at the onset of regeneration (Fig. 10b, c). To test whether zebrafish myomere regeneration is dependent on this signaling, TOR was inhibited using 1 µM rapamycin (Fig. 10d). At 30 dpci, live imaging of the caudal peduncle indicated imperfect restoration of the skin in the rapamycin-treated group compared to the control (Fig. 10e, f). To assess regeneration, cross sections from the same specimen were stained for F-actin and ColXII. In comparison to the control, rapamycin-treated fish displayed a 50% reduction in phalloidin-labeled tissue in the wounded area, suggesting impaired muscle regeneration (Fig. 10g−k). This phenotype was associated with abundant ColXII positive matrix, indicating persistent fibrosis (Fig. 10l). The uninjured side of the cross section was not affected by rapamycin treatment (Supplementary Fig. 8). Thus, the inhibition of TOR signaling prevented muscle regeneration, leading to replacement of skeletal muscle with connective tissue.

## Discussion

Regenerative strategies vary depending on species, age, anatomy, or injury method^35^. Skeletal muscle renewal has been studied in various models, revealing many common cellular and molecular players^66,67^. Here, we identified the processes of myomere regeneration in adult zebrafish. We developed a new cryoinjury method that is suitable for damaging several consecutive myomeres, resulting in muscle degeneration and tissue shrinkage, similar to volumetric muscle loss. In contrast to surgical incision, the integrity of the body remains unaffected, allowing for natural clearance of cryoinjured muscle within 4 days after procedure. Subsequently, in the second phase between 4 and 7 dpci, a provisional matrix is deposited, and the myogenic program becomes activated in the wounded tissue. The third phase, between 7 and 30 dpci, represents progressive expansion and differentiation of myofibers, accompanied by matrix resorption. Finally, the termination phase involves refinement of the compartmental boundary between slow- and fast-twitch muscles. However, some intermingling fibers persisted even at 45 dpci, suggesting imperfect restoration of separation between the muscle compartments. Taken together, myomere regeneration can be described as a sequence of four phases, namely, muscle degeneration, myogenic program activation, muscle restoration, and slow/fast muscle remodeling.

Cryoinjury is defined as controlled damage to tissue by the precise application of extreme cold. Freezing and thawing rupture the cells as a consequence of intracellular fluid ice crystal formation and protein destruction^68^. In mice, freeze injury gives rise to a “dead zone”, devoid of viable cells^69^. The recovery involves directional migration and infiltration of different cell populations leading to regeneration in approximately one month^70^. Autophagy is involved in the clearance of misfolded proteins and disrupted organelles. In our study, the massive accumulation of p62 suggests that the last steps of the pathway could be impaired, contributing to progressive cell death^47,48^. Consequently, tissue degeneration was accompanied by infiltration of immune cells. First, neutrophils invade the damaged area, and as they retract after wound clearance, macrophages accumulate in the regeneration zone. The significance of this switch might be related not only to the clearance of debris but also to the mechanisms activating satellite-like cells in the muscle. Indeed, in mice and zebrafish, the stimulation of normally quiescent stem cells is triggered by microenvironmental changes released by infiltrating macrophages^7,71^. In addition to immune factors, the provisional matrix might modulate the niche of stem and progenitor cells^72,73^. This study revealed that ColXII is rapidly deposited in the wound. ColXII proteins do not assemble into rigid fibrils but form flexible bridges between matrix fibers, facilitating cell migration and tissue remodeling^74^. ColXII has been previously associated with organ regeneration in zebrafish^43,44,75–77^. In the myomere, ColXII-producing cells might derive from disrupted myosepta, which are fasciae between myomeres. Thus, the modulation of the extracellular environment might facilitate muscle regeneration.

In this study, myogenic precursors were traced using the Pax7 antibody to label satellite cells, whereas MyoD1 immunofluorescence served to detect committed myoblasts or myocytes. This double immunofluorescence in combination with F-actin and DAPI staining identified the overlapping stages of myogenesis from undifferentiated stem cells to myofibers^23,30,32,78^. The proliferative phase coincided with the expansion of Pax7-positive cells between 4 and 7 dpci. At 10 dpci, cytoplasmic MyoD1 was detected in immature myofibers. The significance of the nonnuclear localization of MyoD1 warrants further study. Altogether, skeletal muscle regeneration after cryoinjury involves Pax7 and MyoD1 expression in myogenic progenitors in zebrafish.

In fish, satellite cell-dependent myofiber regeneration has been reported in the context of stab injury and laser ablation ^4,7^. Furthermore, electric fish (*S. macrurus*) can repair the muscle after tail amputation through the activation of muscle stem cells^79^. Nevertheless, a satellite cell-independent mechanism has been observed after partial resection of the extraocular muscle in zebrafish^37^. Indeed, myofibers are postmitotic, but they can revert into proliferative myogenic precursors in certain nonamniotic vertebrates, such as newts and zebrafish^38,80,81^. Thus, zebrafish might be able to use alternative regenerative strategies in an organ-dependent manner.

The lateral musculature of the fish includes several types of muscle. This study identified new markers, CT3 (Tnnt2) and N2.261 (Myh7 variant), labeling slow myofibers in the lateral superficial muscle. These markers are helpful to determine the molecular properties of myofibers. We determined that the *smyhc1* promoter of the transgenic line *Tg(smyhc1:LY-Tomato)* is activated in the outermost layers of the Tnnt2-positive compartment of the myomere, suggesting heterogeneity among slow fibers. This result is not surprising, given that the fast-twitch muscle compartment includes regions with intermediate-twitch myofibers^9,15,18,82^. The distinction between different subpopulations of skeletal muscles warrants further investigation. In zebrafish larvae, *smyhc1* is required for slow muscle fiber growth^57^. Importantly, our study established that *smyhc1* was activated in newly formed F-actin-negative myofibers, providing a reporter of undifferentiated muscle during regeneration. This would be consistent with the developmental program, in which slow muscles develop earlier than fast muscles in vertebrates^45,83–85^. This transgenic tool might offer further possibilities to dissect the molecular profile of immature cells during myomere restoration.

The molecular signals for the activation of myogenesis might involve TOR signaling, as inhibition of this pathway impaired regeneration. In mice, TOR signaling is essential for the regulation of myogenic gene expression in muscle stem cells^86–90^, suggesting a conserved role of this pathway among vertebrates. In adult zebrafish, TOR is required for heart and retina regeneration^91,92^. Further studies will elucidate the molecular mechanisms underlying the rapamycin-mediated impairment of muscle regeneration.

Human muscle tissues can regenerate after various traumas, such as ischemia, incisions, or crush injuries. However, traumatic muscle loss and chronic muscle degeneration cannot be repaired by satellite cells, resulting in functional impairment^93,94^. In this context, studies of scarless regeneration of the skeletal muscle in zebrafish might contribute to new perspectives relevant for regenerative medicine.

## Methods

### Animal strains and procedures

Wild-type fish were AB (Oregon). Transgenic lines were *Tg(smyhc1:LY-Tomato)^i261*^59^, *Tg(smyhc1:egfp)^i104*^58^, and *Tg(-2.2mylz2:EGFP)^i135*^45^. For identification of transgenic fish by fluorescence stereomicroscopy, animals were anaesthetized with a buffered solution of 0.6 mM tricaine (MS-222 ethyl-m-aminobenzoate, Sigma-Aldrich) in system water.

Adult zebrafish were of random sex and aged between 4 and 18 months. Before tissue collection, fish were euthanized according to the approved protocol by the cantonal authorization. The fish were incubated for 10 min in water containing 300 mg/L buffered tricaine and chilled afterwards. The caudal peduncle was immediately dissected and fixed. All assays were performed using different animals randomly assigned to experimental groups. The exact sample size (n) was described for each experiment in the figure legends and was chosen to ensure the reproducibility of the results.

### Drug treatment

To inhibit the mTOR pathway, rapamycin (Selleckchem) was dissolved in DMSO at a concentration of 10 mM and used at a final concentration of 1 μM in fish water. Fish were pre-treated with 0.1% DMSO in the control or drug solution for one day prior to injury. They recovered without treatment during the first day after cryoinjury and subsequently were kept in treatment until the time of tissue collection.

### Cryoinjury procedure

According to the cantonal regulation, one hour prior to injury, analgesia was achieved by immersing the animals in 0.6 mM lidocaine (2-diethylamino-N-(2,6-dimethyl-phenyl)-acetamide, Sigma-Aldrich) in water. Fish were subsequently anaesthetized with a buffered solution of 0.6 mM tricaine (MS-222 ethyl-m-aminobenzoate, Sigma-Aldrich) in system water and placed left side up on a damp sponge. Skeletal muscle cryoinjury was performed with a stainless steel cryoprobe (custom manufactured at the university workshop): the overall weight was 9 g, and the terminal spatula was 1 mm thick, 5 mm broad and 6 mm long with an inward curved edge, corresponding to the shape of the zebrafish body. The 14 cm long handle was inserted in a plastic pipette tip for handling. The cryoprobe was precooled in liquid nitrogen and placed for six seconds on the skin of the fish, perpendicular to the surface of the left side, between the anal and caudal fins. The weight of the probe is sufficient as pressure, and any additional force applied could lead to mortality. Immediately after the procedure, fish were transferred back to the system water and continuously monitored until they restarted swimming. Zebrafish usually resumed breathing within a few seconds after the transfer. The details of the procedure are described in a video publication^42^.

### Collection and fixation of the tissue

After euthanasia, the caudal peduncle was resected with surgical scissors. Skeletal muscle samples were fixed overnight at 4 °C in water with 4% paraformaldehyde (PFA). The fixation step was followed by a 10 min wash in phosphate buffered saline (PBS) and equilibration at 4 °C in 30% sucrose for 1 to 2 days. Samples were then embedded in tissue freezing media (Tissue-Tek O.C.T.; Sakura), quickly frozen on dry ice and stored overnight or longer at −80 °C. Finally, tissues were cut with a cryostat at a thickness of 25 μm. Sections were collected on Superfrost Plus slides (Fisher), dried at room temperature (RT) for approximately one hour and stored at −20 °C in tight boxes.

### Histological analysis

For histological staining, aniline blue, acid fuchsin and orange-G (AFOG) were utilized, as previously described^95^. In summary, sections were fixed in 10% formalin for 15 min and washed in PBST (PBS + 0.3% Triton-X) for 10 min. Next, slides were incubated in preheated Bouin’s fixative (Reactolab, Servion, Switzerland) for 2.5 h at 56 °C and one hour at room temperature. The slides were washed for 20 min in tap water and then transferred into 1% phosphomolybdic acid for 5 min. They were rinsed with distilled water and incubated for 4 min with AFOG solution (3 g acid Fuchsin, 2 g Orange G, 1 g Aniline Blue, 200 mL acidified distilled water pH 1.1) followed by washing steps with distilled water to remove excessive staining. Finally, sections were dehydrated in a graded series of ethanol, passed through xylol, and mounted with Entellan (107961, Merck Millipore). The imaging of stained sections was performed using a Zeiss Axioplan2 microscope.

### Immunofluorescence analysis

Skeletal muscle slides were encircled with an ImmEdge Pen (Vector Laboratories) and left to dry for 10 min at room temperature (RT) to create a hydrophobic barrier. To rehydrate tissues and permeabilize membranes, slides were washed for 10 min in a coplin jar containing 0.3% Triton-X in PBS (PBST). To prevent nonspecific binding, blocking solution containing 5% goat serum in PBST was applied in a humid chamber for two hours at RT. Subsequently, sections were covered with 200 μL of primary antibody diluted in blocking solution and incubated overnight at 4 °C in the same humid chamber. The next day, slides were washed for one hour at RT with PBST in coplin jars before being transferred to a humid chamber again for a two-hour incubation at RT with secondary antibodies diluted in blocking solution. In the case of the anti-guinea pig antibody in combination with other secondary antibodies, a 15 min wash in PBST followed by a similar independent incubation step was performed to avoid cross-reaction between secondary antibodies. A last washing step was performed with PBST in coplin jars for one hour at RT, and slides were then mounted in 90% glycerol in 20 mM Tris pH 8 with 0.5% N-propyl gallate.

In case of PCNA immunostaining, antigen retrieval was performed as previously described^91^. Briefly, the sections were permeabilized for 10 min in 0.3% PBST and then subjected to a heat-mediated epitope retrieval step. Specifically, slides were transferred to coplin jars containing 10 mM sodium citrate dihydrate solution (Sigma-Aldrich, W302600) and 0.05% Tween-20 (Sigma, T2700). They were placed in a domestic pressure cooker that was preheated on a hot plate. As soon as the cooker has reached full pressure, time of boiling was 6 min. After cooling in a sink, sections were washed in 0.3% PBST for 5 min, air dried and processed according to the staining protocol.

The following primary antibodies were used: guinea pig anti-Col12a at 1:100 (kindly provided by F. Ruggiero, Lyon, France)^96^, mouse anti-Troponin T2 (Tnnt2) at 1:100 (CT3, developed by Lin, J.J.-C., obtained from Developmental Studies Hybridoma Bank), chicken anti-mCherry/Tomato (Cat#: MCherry-0020 Aves Labs, Inc.) at 1:200, and guinea pig anti-p62/SQSTM1 (Progen, GP62-C) at 1:200, mouse IgG1 anti-Myosin heavy chain (MYH7 or embCMHC) at 1:50 (N2.261, developed by H.M. Blau, obtained from Developmental Studies Hybridoma Bank), mouse IgG1 anti-Pax7 at 1:5 (PAX7, developed by A. Kawakami, obtained from Developmental Studies Hybridoma Bank), mouse IgG2a anti-Proliferating Cell Nuclear Antigen (PCNA) at 1:100 (Code#: M0879, Dako), rabbit anti-Mpx at 1:200 (Cat#: GTX128379, GeneTex), chicken anti-L-Plastin at 1:200 (kindly provided by P. Martin, Bristol)^51^, rabbit anti-Phospho-S6 ribosomal protein (p-rpPS6) at 1:200 (Cat#: 5364, Cell Signaling Technology), rabbit anti-Myl7 at 1:500 (GTX128346, GeneTex), rabbit anti MyoD1 at 1:200 (GTX128129, GeneTex) and chicken anti-GFP at 1:500 (GFP-1010, AVES labs). The secondary antibodies were Alexa conjugated (Jackson ImmunoResearch Laboratories). Phalloidin-Atto-565 (94072), Phalloidin-CruzFluor-405 (sc-363790, Santa Cruz Biotechnology) and Phalloidin-CruzFluor-488 (sc-363791, Santa Cruz Biotechnology) were used at 1:500 to label actin filaments. DAPI (Sigma) was used at 1:2000 to detect nuclei.

### Confocal fluorescence microscopy

Slides were imaged (1024×1024 pixels) with either Leica SPEII microscope using ACS Apochromat 20x/0.6 NA oil lens or an ACS Apochromat 40x/1.15 NA oil lens or Leica SP5 microscope using 20x/0.7 immersion lens. Multiple tiles were taken in picture and then merged to get the whole tissue in picture. Microscopes were set up to avoid any bleed through between channels. Images were optimized using ImageJ home-made macro. Especially, for expected nuclei signal, background was removed with rolling ball radius of 5 pixels. Skin and dermal scales emanating fluorescence outside the muscle were erased from images using Adobe Photoshop for clarity.

### In-situ hybridization

The following forward (F) and reverse (R) primers were used to synthesize the template for the probes: *smhyc1* (NM_001020507.1) F: 5’-GTCAAGGATTCCCAAATGCAAC-3’ and R: 5’-TGGCTTCACAACAAACAAACTC -3’; LY-Tomato (AY678269.1) F: 5’-GGGCGAGGAGGTCATCAAAGAG-3’ and R: 5’-GGCCATGTTGTTGTCCTCGG-3’.

To synthesize the anti-sense probes, a sequence of T3 RNA polymerase promoter was added to the 5’ end of the reverse primers, whereas to generate sense probes, a sequence of T7 TNA polymerase promoter was added to the 3’ end of the forward primers. The digoxigenin-RNA labeling mix (Roche) was used to generate the probe, which after purification was dissolved in hybridization solution and stored at −20 °C.

Caudal peduncles were fixed in 4% PFA overnight at 4 °C, washed twice in PBS, dehydrated in a series of 30 min incubations in 25%, 50%, 75% of methanol/PBS at room temperature, and stored in 100% methanol overnight or longer at 4°C. Then, the specimens were rehydrated in the inverse concentration order of methanol/PBS solutions. The rehydrated caudal peduncles were again dehydrated in 30% sucrose for at least 2 h, embedded in O.C.T. medium in molds and cryosectioned at the thickness of 25 µm, as for immunofluorescence. Before use, slides were brought to room temperature for 10 min, and processed according to our standard protocol described previously for the fin and the heart^77,97^. After completion of in situ hybridization, florescent staining was performed for Tnnt2 to visualize slow muscle and DAPI to label all nuclei. After immunofluorescence staining, slides were mounted in 90% glycerol in 20 mM Tris pH 8 with 0.5% N-propyl gallate. Brightfield images were taken using a Zeiss microscope and Axiovision camera using a 10x lens, whereas the corresponding fluorescence images were taken using a Leica SPE-II confocal microscope using a 20x lens.

### Video analysis of swimming fish

Video recording of adult zebrafish was conducted between 14:00 and 16:00. The tank was 20 × 10 x 12 cm with transparent walls. The camera (Sony) was fixed above the tank. Five zebrafish were habituated for 1 min, followed by filming for 10 min. Swimming parameters were analyzed with EthoVision software (Noldus).

### Quantifications and statistical analysis

Each biological replicate (*N*) corresponds to one fish. For transverse sections, 2 to 3 nonadjacent sections were analyzed that were interspaced from each other by a minimum of 200 μm within the organ. Error bars correspond to the standard error of the mean (SEM). ImageJ was used to quantify the surface distribution of proteins, to count the number of nuclei and to determine the number of nuclei positive for multiple proteins.

To measure the immunostaining area, the brightness parameter was first adjusted to eliminate as much of the background as possible. The different regions of interest are drawn by hand and isolated from the rest of the image. First, the area of the entire region of interest is measured (Set Measurements → activate “Area” and “Limit to Threshold”, Measure). Then, using the channel of interest (F-actin, ColXII, myosin), a mask is obtained using an appropriate threshold. The mask resulting from this manipulation may present “holes” inside a masked zone, which gives a false measurement of the mask surface. To obtain the surface area of the masked zone without taking these holes into account, the “watersheld” tool is used, making it possible to form masked zones that no longer contain “holes”. Finally, the total area of the masks is measured (Set Measurements → x Area, Analyze Particle [Size :0-Infinity, Circularity : 0.00-1.00]). The percentage of an area marked by a protein corresponds to the second measurement divided by the first measurement.

For colocalization analysis of a nuclear signal, the background was first removed using a rolling ball with a radius of 5 pixels (process→subtract background), and then the brightness parameter was adjusted. Next, the wound regions or its corresponding control side was hand-drawn and isolated from the rest of the image. All nuclei were first counted using the DAPI channel. A mask is obtained from the DAPI signal using an appropriate threshold. The mask is enhanced by separating the round elements from each other using “watershed” and “fill holes”. Finally, nuclei are counted using ITCN (Image-based Tool for Counting Nuclei, https://bioimage.ucsb.edu/docs/automatic-nuclei-counter-plugin-imagej; width = 10, minimum distance = 5, threshold = 0). Secondly, the number of nuclei positive at another channel is obtained as follows. Using the “colocalization” plug-in (https://imagejdocu.list.lu/plugin/analysis/colocalization_analysis_macro_for_red_and_green_puncta/start; ratio = 40; threshold_channel_1 = 20, threshold_channel_2 = 20, display = 255), only nuclei visible in another channel are isolated and counted using ITCN (same parameter as above). To count the number of positive nuclei for several channels, the mask obtained from a previous colocalization is used for the next colocalization instead of the DAPI mask. Java custom script used for the analysis are available on demand. Graphics and statistical analyses were made with GraphPad Prism software or on R (R version 4.3.0, tidyverse version 2.0.0). Statistical plots were performed with GraphPad Prism software.

### Ethics approval

This study complied with all relevant ethical regulations. Zebrafish were bred, raised, and maintained in accordance with the FELASA guidelines^98^. The animal housing and all experimental procedures were approved by the cantonal veterinary office of Fribourg, Switzerland.

### Reporting summary

Further information on research design is available in the Nature Research Reporting Summary linked to this article.

### Supplementary information

Supplemental Material

reporting summary

Movie 1

Movie 2

Movie 3

## Data Availability

The authors declare that all data supporting the findings of this study are available within the article and its Supplemental Material files, or from the corresponding author upon request.
