# Supplementary material for: Skeletal muscle regeneration after extensive cryoinjury of caudal myomeres in adult zebrafish
Source: NPJ Regen Med. 2024 Feb 20;9:8. doi: 10.1038/s41536-024-00351-5 (PMC10879182; doi:10.1038/s41536-024-00351-5)
Supplement: Supplementary file 1 — Supplemental Material [file 41536_2024_351_MOESM1_ESM.pdf]

Supplementary Material

Skeletal muscle regeneration after extensive cryoinjury of caudal myomeres in adult zebrafish

Hendrik Oudhoff, Vincent Hisler, Florian Baumgartner, Lana Rees, Dogan Grepper and Anna Jaźwińska *

Department of Biology, University of Fribourg, Chemin du Musée 10, 1700 Fribourg, Switzerland

* corresponding author

Email: anna.jazwinska@unifr.ch

Supplementary Movies 1-3:

Movie 1_uninjured

Movie 2 _1 dpci

Movie 3_10 dpci

Representative film clips from 10 min long videos recorded at the indicated time points post-cryoinjury. At each condition, 5 fish were habituated for 1 min and then filmed for 10 min for analysis. Tracking of swimming patterns was performed with the EthoVision software (Noldus). Quantification of the results is shown in (**Fig. 9**).

Supplementary Fig. 1- Fig. 8

Supplementary Fig. 1

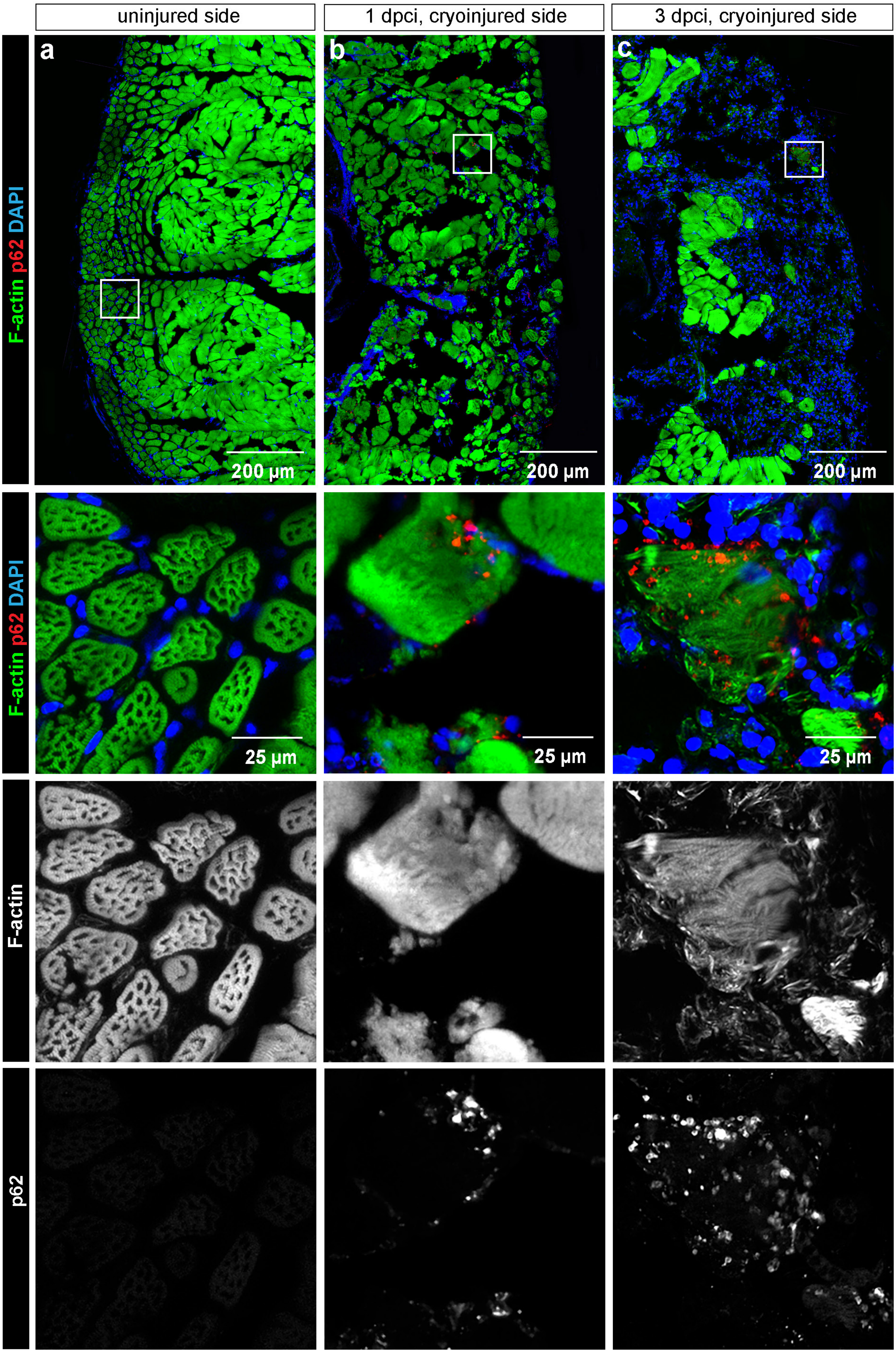

Supplementary Fig. 1. The accumulation of the selective autophagy receptor p62 in degenerating muscle after cryoinjury.

Cross sections were stained for p62 (receptor of selective autophagy, red), phalloidin (F-actin, green) and DAPI (nuclei, blue). N = 3 fish per time-point.

a In uninjured side of fish at 3 dpci, no p62 is detected in the cross sectioned myofibers.

b At 1 dpci, the cryoinjured side contains disrupted F-actin staining. p62 puncta are present in damaged myofibers.

c At 3 dpci, the cryoinjured side contains few myofibers, suggesting the clearance of F-actin. The remaining myofibers display abnormal distribution of F-actin and accumulation of p62 puncta, some of which are in the form of vesicles.

Supplementary Fig. 2

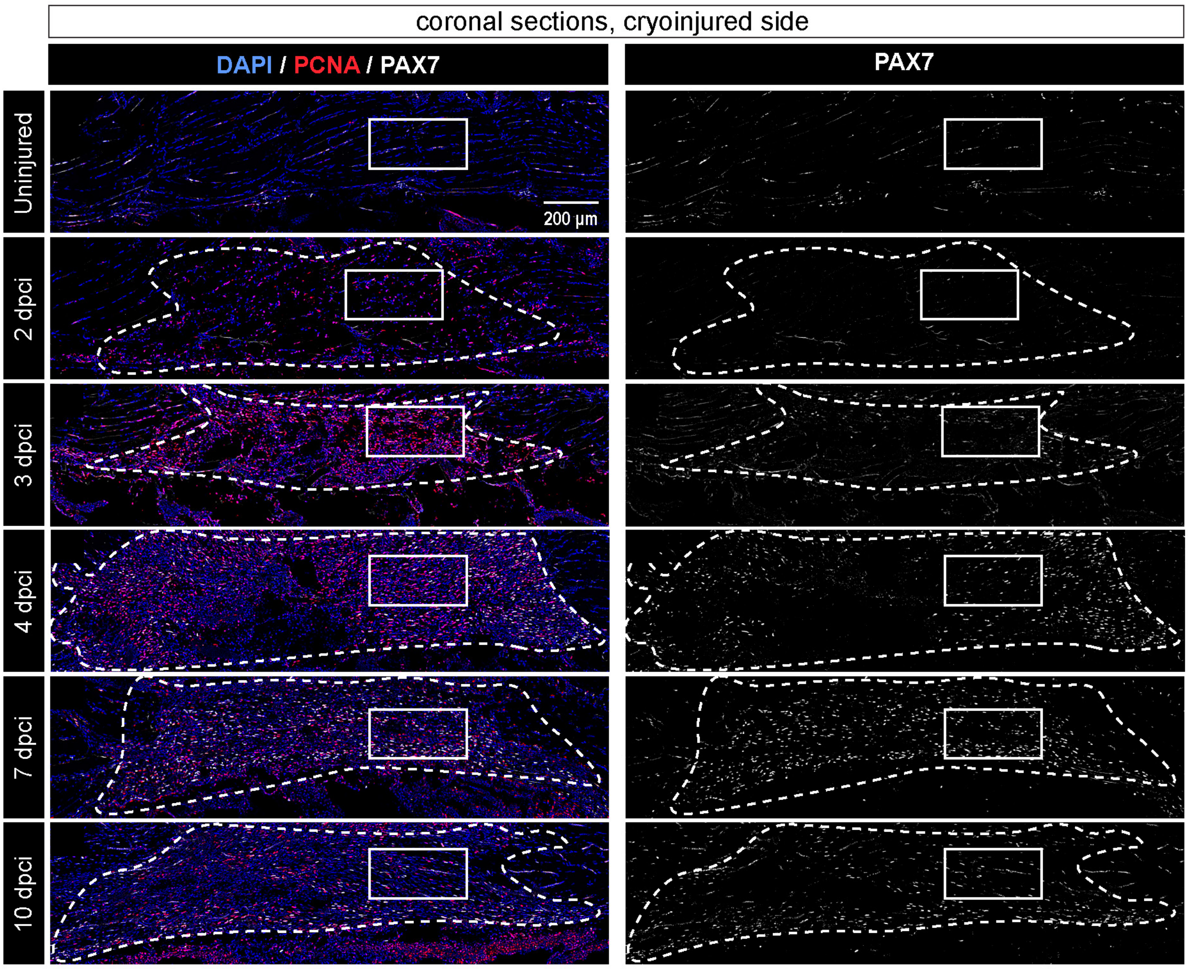

Supplementary Fig. 2. PAX7+ satellite cells are activated during initiation of muscle regeneration.

Coronal sections fluorescently stained for four markers, of which the figure displays Pax7 (gray), PCNA (red) and DAPI (blue). The non-displayed channel is muscle staining shown in (**Fig. 3a-f**). The frames depict the areas magnified in (**Fig. 4a**) and quantified in (**Fig. 4b, c**).

Supplementary Fig. 3

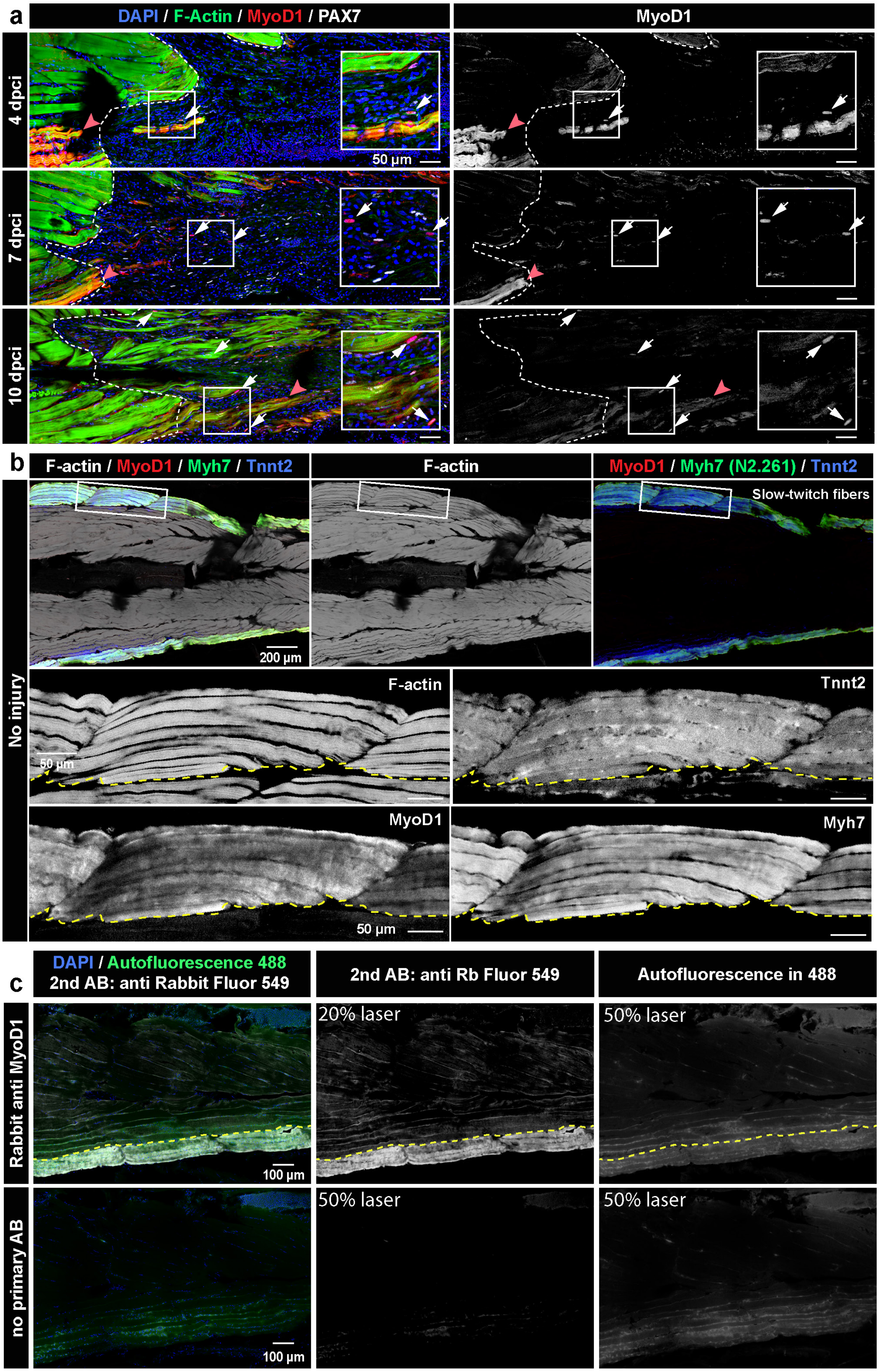

Supplementary Fig. 3. Activation of the myogenic program after wound clearance.

a Immunofluorescence staining of coronal sections for Pax7 and MyoD1, co-labeled with phalloidin for F-actin and DAPI for nuclei. N = 3. MyoD1 is immunodetected in nuclei (white arrows) and cytoplasm of myofibers (red arrowheads).

b Coronal sections of uninjured fish, fluorescently stained for four markers. Several outer layers of myofibers display colocalization of F-Actin, Tnnt2 (CT3 antibody), MyoD1 and Myh7 (N2.261 antibody).

c Test for the specificity of MyoD1 immunostaining, visualized with Alexa 549-congugated secondary antibody, performed on coronal section of uninjured fish. The sections were counterstained only with DAPI to avoid any bleed-through from a neighboring channel. To assess autofluorescence and the confocal microscope setting, the staining was performed with or without the MyoD1 antibody and with different intensities of the relevant laser.

Supplementary Fig. 4

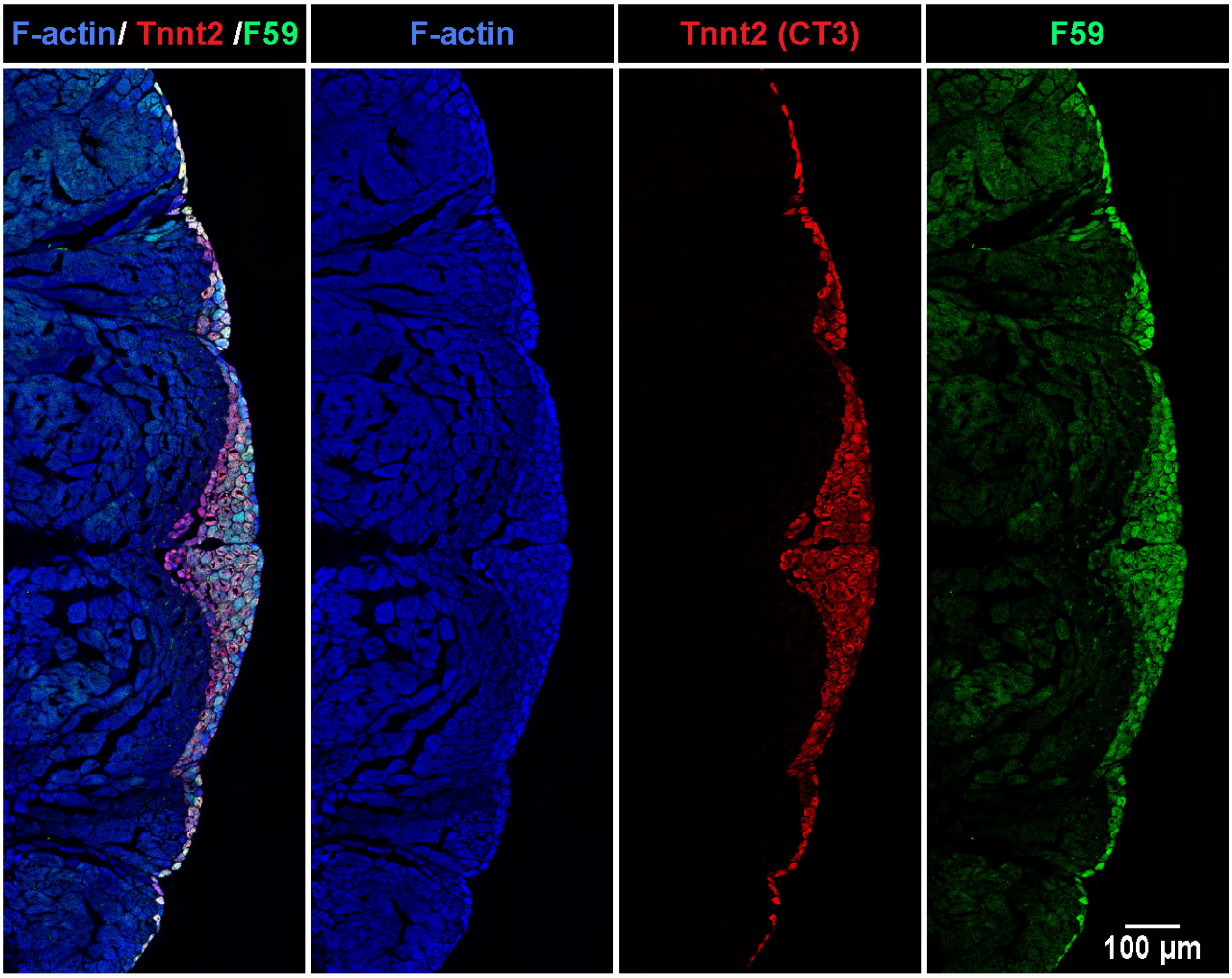

Supplementary Fig. 4. Colocalization between the previously characterized marker of slow muscle, F59 antibody, and the Tnnt2 expression detected with the CT3 antibody.

Cross section of uninjured fish was fluorescently stained with the CT3 and F59 antibodies and phalloidin (F-actin). The image displays one side of the body. N = 3 sections.

Supplementary Fig. 5

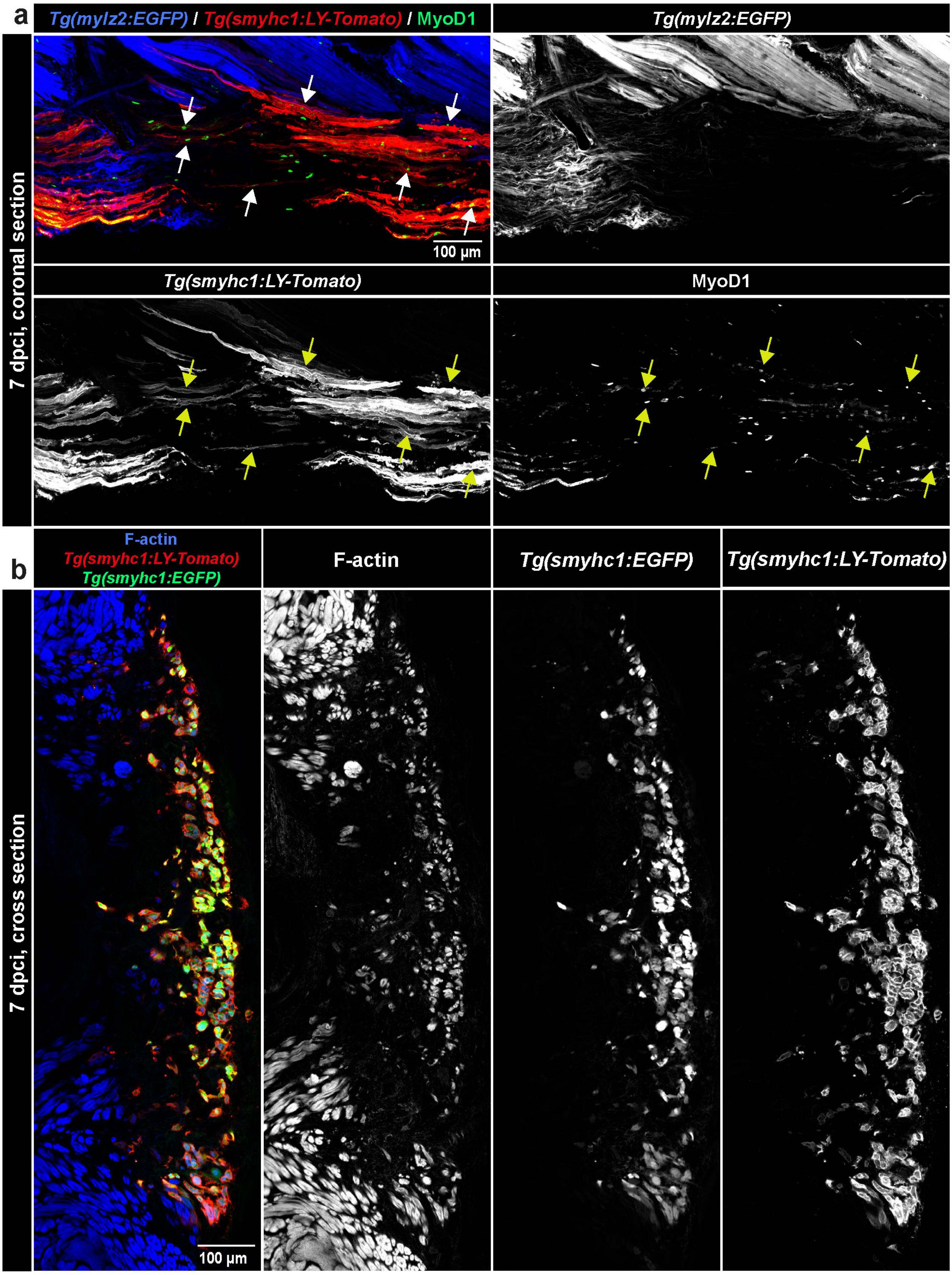

Supplementary Fig. 5. The transgenic reporter *smyhc1:LY-Tomato* is activated in regenerating myofibrils in the superficial and profound muscles.

a Coronal sections of the *mylz2:EGFP* and *smyhc1:LY-Tomato* double transgenic fish at 7 dpci, stained with Tomato/Cherry and MyoD1 antibodies. The image displays the cryoinjured tissue. LY-Tomato is detected in the superficial and profound portion of the regenerating muscle. This expression

is associated with a nuclear MyoD1 localization (arrows), suggesting that both markers demarcate newly formed immature myofibers. N = 3.

b Cross section of the peri-injury zone (wound margin) in *smyhc1:LY-Tomato* and *smyhc1:EGFP* double transgenic fish, co-stained for LY-Tomato and F-actin. At 7 dpci, EGFP and LY-Tomato are expressed in similar cells corresponding to newly formed myofibers. The subcellular localization of both proteins is not overlapping because EGFP is cytoplasmic whereas LY-Tomato is localized at the plasma membrane. N = 3.

Supplementary Fig. 6

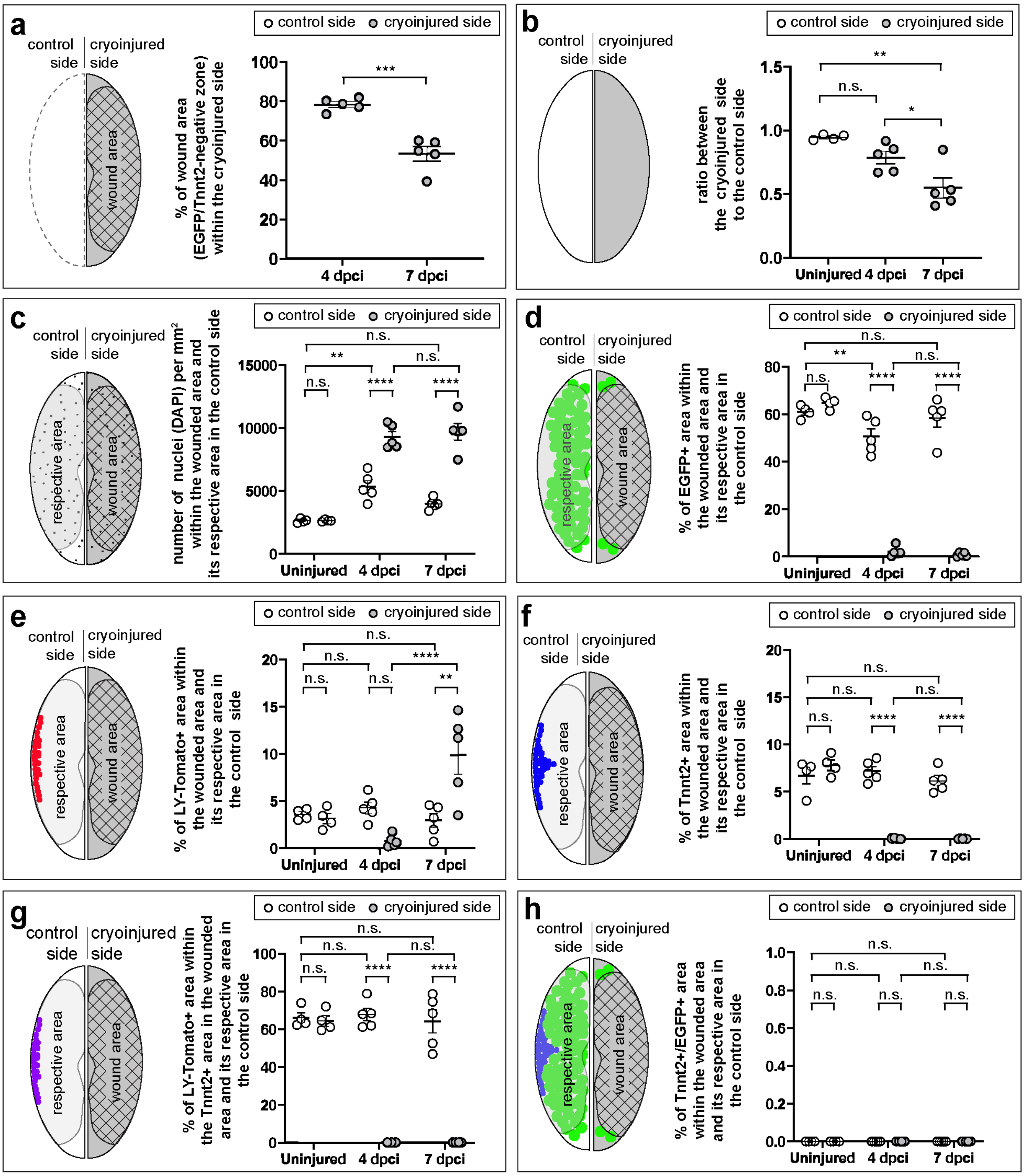

Supplementary Fig. 6. Quantification of image data representatively shown in Fig. 7.

a-h The analyzed parameters are schematically illustrated on the left side to the graph. Control side is considered as the opposite side to the cryoinjury side. “Wound area” is determined by the loss of *mylz2:EGFP* and Tnnt2 expression the injured flank. “Respective area” is an approximate mirror-image area of the wound at the control side. The color of specific markers corresponds to the staining in **Fig. 7**.

a Quantification of the wound size, calculated as a percentage of *mylz2:EGFP* /Tnnt2 negative area relative to the cryoinjured side at 4 and 7 dpci.

b Comparison of right and left sides of the body, calculated as ratios between the lateral areas in fish with no injury, and ratios between the cryoinjured side relative to the control side in fish at 4 and 7 dpci.

c Quantification of the nuclear density, calculated as number of DAPI stained nuclei per mm^2 of the wounded area and its respective control side.

d-f Quantification of specific muscle markers: *mylz2:EGFP* **(d)**, *smyhc1:LY-Tomato* **(e)** and Tnnt2 **(f)**, calculated as percentage of fluorescent area relative to the wounded area and its respective control side.

g Quantification of the overlap between *smyhc1:LY-Tomato* and Tnnt2, calculated as percentage of *smyhc1:LY-Tomato* positive area within Tnnt2 positive area in the wound and to its respective control side. *smyhc1:LY-Tomato* is expressed in a portion of the Tnnt2-immunoreactive region.

h Quantification of the overlap between Tnnt2 positive and *mylz2:EGFP* positive area in the wound and its respective control side. No co-expression is detected at any conditions.

Uninjured fish: N = 4 (average of 3 sections per fish); cryoinjured fish: N = 5 (average of 3 sections per fish). Statistical test for (**a**): unpaired two-tailed Student’s t-test, for (**b**): one-way ANOVA with Tukey’s multiple comparisons test, for (**c-h**): two-way ANOVA with Tukey’s multiple comparisons test). For all panels: error bar, SEM; **** P<0.0001, ***P<0.001, ** P<0.01, *P<0.05.

Supplementary Fig. 7

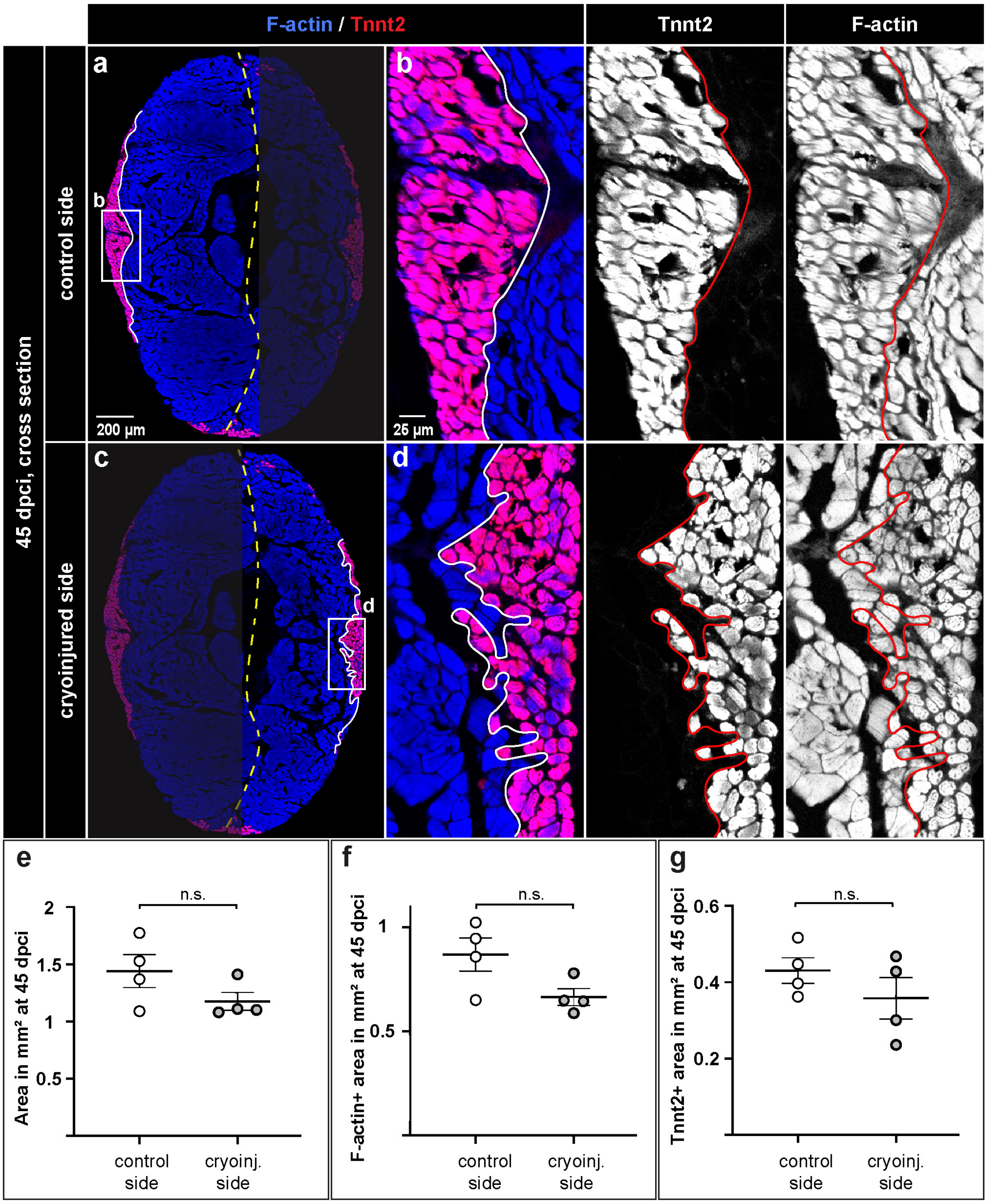

Supplementary Fig. 7.

Imperfect restoration of the separation between the slow and fast muscle compartments.

a-d Cross section of the caudal peduncle of wild type fish at 45 dpci, stained with phalloidin to detect F-actin (blue) and the Tnnt2 antibody (red). Yellow dashed line in (A and C) delineates the vertical midline. (**a**) The cryoinjured half (right on the image) is masked to focus on the control side that is magnified in (**b**). (**c**) The control half (left on the image) is masked to present the cryoinjured side that is magnified in (**d**). Lines in **(b** and **d)** depict the border between Tnnt2 positive and negative muscle.

e-g Quantification of the muscle area in the control and cryoinjured side at 45 dpci. **(e)** Total area of the left and the right side. **(f)** F-actin positive area. **(g)** Tnnt2 positive area. Cryoinjured fish: N = 4 (average of 3 sections per fish); error bar, SEM; n.s., not significant, P > 0.05, unpaired two-tailed Student's t-test.

Supplementary Fig. 8

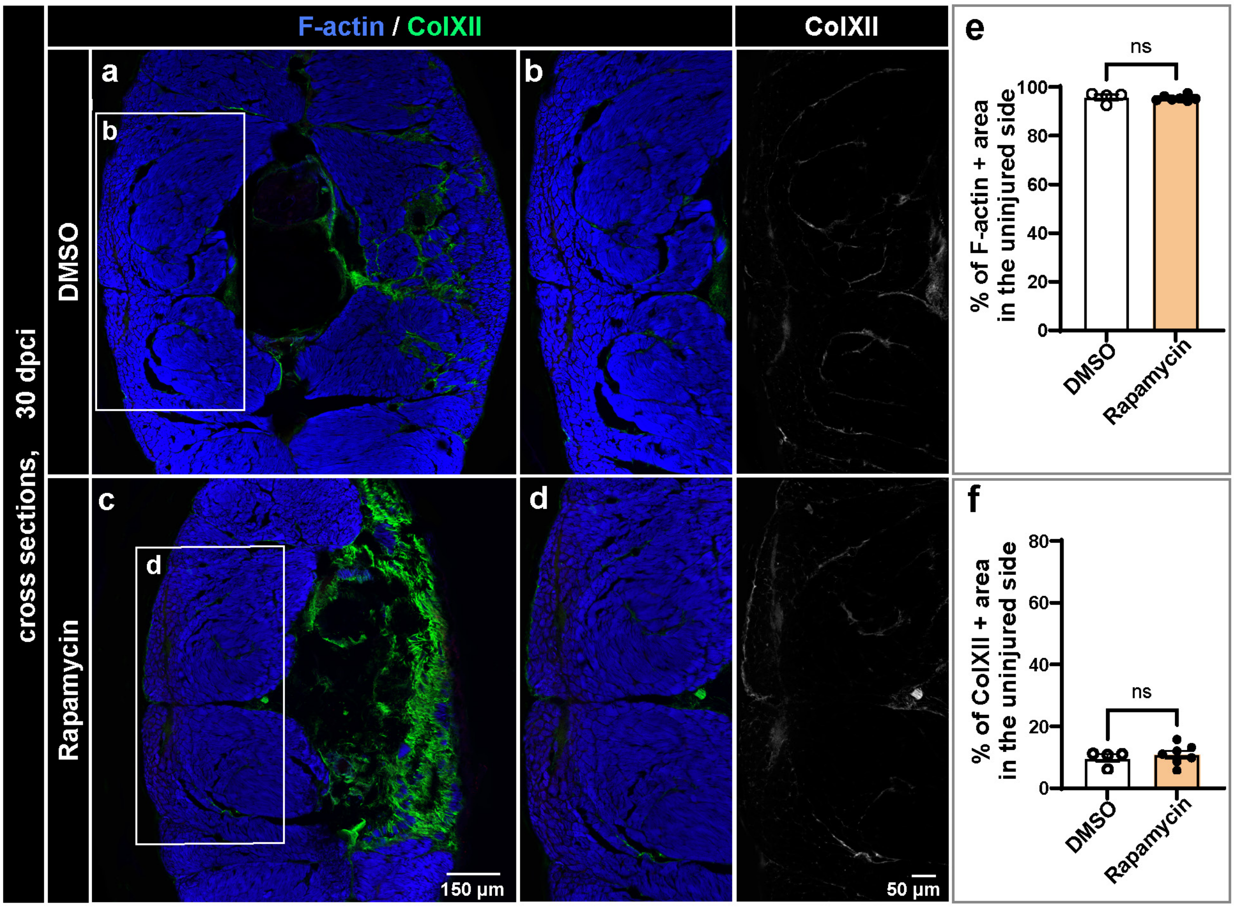

Supplementary Fig. 8. Rapamycin treatment does not alter the uninjured side of the fish.

a, c The same images as in (**Fig. 10g, i**), but with a frame on the uninjured side to demarcate the enlarged area shown to the right.

b, d Magnified areas of the uninjured side.

e, f Quantification of F-actin and ColXII does not indicate any effects of Rapamycin treatment on both markers within the uninjured side. DMSO group: N = 4; Rapamycin group: N = 7. Error bar, SEM. Unpaired two-tailed Student’s t-test.
