# Supplementary material for: Skeletal muscle regeneration after extensive cryoinjury of caudal myomeres in adult zebrafish
Source: NPJ Regen Med. 2024 Feb 20;9:8. doi: 10.1038/s41536-024-00351-5 (PMC10879182; doi:10.1038/s41536-024-00351-5)
Supplement: Supplementary file 2 — reporting summary [file 41536_2024_351_MOESM2_ESM.pdf]

nature portfolio

Corresponding author(s): Anna Jazwinska

Last updated by author(s): 2023.11.28

Reporting Summary

Nature Portfolio wishes to improve the reproducibility of the work that we publish. This form provides structure for consistency and transparency in reporting. For further information on Nature Portfolio policies, see our Editorial Policies and the Editorial Policy Checklist.

Statistics

For all statistical analyses, confirm that the following items are present in the figure legend, table legend, main text, or Methods section.

n/a	Confirmed	
☐	☒	The exact sample size (*n*) for each experimental group/condition, given as a discrete number and unit of measurement
☐	☒	A statement on whether measurements were taken from distinct samples or whether the same sample was measured repeatedly
☐	☒	The statistical test(s) used AND whether they are one- or two-sided<br>*Only common tests should be described solely by name; describe more complex techniques in the Methods section.*
☐	☒	A description of all covariates tested
☐	☒	A description of any assumptions or corrections, such as tests of normality and adjustment for multiple comparisons
☐	☒	A full description of the statistical parameters including central tendency (e.g. means) or other basic estimates (e.g. regression coefficient) AND variation (e.g. standard deviation) or associated estimates of uncertainty (e.g. confidence intervals)
☐	☒	For null hypothesis testing, the test statistic (e.g. *F*, *t*, *r*) with confidence intervals, effect sizes, degrees of freedom and *P* value noted<br>*Give P values as exact values whenever suitable.*
☒	☐	For Bayesian analysis, information on the choice of priors and Markov chain Monte Carlo settings
☒	☐	For hierarchical and complex designs, identification of the appropriate level for tests and full reporting of outcomes
☒	☐	Estimates of effect sizes (e.g. Cohen's *d*, Pearson's *r*), indicating how they were calculated

Our web collection on statistics for biologists contains articles on many of the points above.

Software and code

Policy information about availability of computer code

Data collection	The imaging of histological sections was performed using a Zeiss Axioplan2 microscope using 10x objective.<br>Confocal fluorescence microscopy was performed using either Leica SPEII microscope with ACS Apochromat 20x/0.6 NA oil lens or an ACS Apochromat 40x/1.15 NA oil lens or Leica SP5 microscope using 20x/0.7 immersion lens.
Data analysis	Each biological replicate (n) corresponds to one fish. For transverse sections, 2 to 3 nonadjacent sections were analyzed that were interspaced from each other by a minimum of 200 µm within the organ. Error bars correspond to the standard error of the mean (SEM). ImageJ was used to quantify the surface distribution of proteins, to count the number of nuclei and to determine the number of nuclei positive for multiple proteins. In the latter case, the Image-based Tool for Counting Nuclei (ITCN) was used. Java custom script used for the analysis are available on demand. Graphics and statistical analyses were made with GraphPad Prism software or on R (R version 4.3.0, tidyverse version 2.0.0). Statistical plots were performed with GraphPad Prism software.

For manuscripts utilizing custom algorithms or software that are central to the research but not yet described in published literature, software must be made available to editors and reviewers. We strongly encourage code deposition in a community repository (e.g. GitHub). See the Nature Portfolio guidelines for submitting code & software for further information.

Data

Policy information about availability of data

All manuscripts must include a data availability statement. This statement should provide the following information, where applicable:

- Accession codes, unique identifiers, or web links for publicly available datasets
- A description of any restrictions on data availability
- For clinical datasets or third party data, please ensure that the statement adheres to our policy

The authors declare that all data supporting the findings of this study are available within the article and its Supplemental Material files, or from the corresponding author upon request.

Research involving human participants, their data, or biological material

Policy information about studies with human participants or human data. See also policy information about sex, gender (identity/presentation), and sexual orientation and race, ethnicity and racism.

Reporting on sex and gender	*Use the terms sex (biological attribute) and gender (shaped by social and cultural circumstances) carefully in order to avoid confusing both terms. Indicate if findings apply to only one sex or gender; describe whether sex and gender were considered in study design; whether sex and/or gender was determined based on self-reporting or assigned and methods used. Provide in the source data disaggregated sex and gender data, where this information has been collected, and if consent has been obtained for sharing of individual-level data; provide overall numbers in this Reporting Summary. Please state if this information has not been collected. Report sex- and gender-based analyses where performed, justify reasons for lack of sex- and gender-based analysis.*
Reporting on race, ethnicity, or other socially relevant groupings	*Please specify the socially constructed or socially relevant categorization variable(s) used in your manuscript and explain why they were used. Please note that such variables should not be used as proxies for other socially constructed/relevant variables (for example, race or ethnicity should not be used as a proxy for socioeconomic status). Provide clear definitions of the relevant terms used, how they were provided (by the participants/respondents, the researchers, or third parties), and the method(s) used to classify people into the different categories (e.g. self-report, census or administrative data, social media data, etc.) Please provide details about how you controlled for confounding variables in your analyses.*
Population characteristics	*Describe the covariate-relevant population characteristics of the human research participants (e.g. age, genotypic information, past and current diagnosis and treatment categories). If you filled out the behavioural & social sciences study design questions and have nothing to add here, write "See above."*
Recruitment	*Describe how participants were recruited. Outline any potential self-selection bias or other biases that may be present and how these are likely to impact results.*
Ethics oversight	*Identify the organization(s) that approved the study protocol.*

Note that full information on the approval of the study protocol must also be provided in the manuscript.

Field-specific reporting

Please select the one below that is the best fit for your research. If you are not sure, read the appropriate sections before making your selection.

☒ Life sciences ☐ Behavioural & social sciences ☐ Ecological, evolutionary & environmental sciences

For a reference copy of the document with all sections, see nature.com/documents/nr-reporting-summary-flat.pdf

Life sciences study design

All studies must disclose on these points even when the disclosure is negative.

Sample size	Sample sizes were determined based on expected effect size of interest with a minimum of 3 biological replicates for any given experiment.
Data exclusions	No data were excluded.
Replication	At least two technical replicates (tissue sections) in a minimum of 3 biological replicates per condition were performed for all experiments.
Randomization	Animal age, sex and size were randomly assigned to experimental groups.
Blinding	no blinding

Reporting for specific materials, systems and methods

We require information from authors about some types of materials, experimental systems and methods used in many studies. Here, indicate whether each material, system or method listed is relevant to your study. If you are not sure if a list item applies to your research, read the appropriate section before selecting a response.

Materials & experimental systems

n/a	Involved in the study
☐	☒ Antibodies
☒	☐ Eukaryotic cell lines
☒	☐ Palaeontology and archaeology
☐	☒ Animals and other organisms
☒	☐ Clinical data
☒	☐ Dual use research of concern
☒	☐ Plants

Methods

n/a	Involved in the study
☒	☐ ChIP-seq
☒	☐ Flow cytometry
☒	☐ MRI-based neuroimaging

Antibodies

Antibodies used	guinea pig anti-Col12a at 1:100 (kindly provided by F. Ruggiero, Lyon, France)<br>mouse anti-Troponin T (Tnnt2) at 1:100 (CT3, developed by Lin, J.J.-C., obtained from Developmental Studies Hybridoma Bank)<br>chicken anti-mCherry/Tomato (Cat#: MCherry-0020 Aves Labs, Inc.)<br>guinea pig anti-p62/SQSTM1 (Progen, GP62-C)<br>mouse IgG1 anti-Myosin heavy chain (MYH7 or embCMHC) at 1:50 (N2.262, developed by H.M. Blau, obtained from Developmental Studies Hybridoma Bank)<br>mouse IgG1 anti-Pax7 at 1:5 (PAX7, developed by A. Kawakami, obtained from Developmental Studies Hybridoma Bank)<br>mouse IgG2a anti-Proliferating Cell Nuclear Antigen (PCNA) at 1:100 (Code#: M0879, Dako)<br>rabbit anti-Mpx at 1:200 (Cat#: GTX128379, GeneTex)<br>chicken anti-L-Plastin at 1:200 (kindly provided by P. Martin, Bristol)<br>rabbit anti-Phospho-S6 ribosomal protein (p-rpPS6) at 1:200 (Cat#: 5364, Cell Signaling Technology)<br>rabbit anti-Myl7 at 1:500 (GTX128346, GeneTex)<br>rabbit anti MyoD1 at 1:200 (GTX128129, GeneTex)<br>chicken anti-GFP at 1:500 (GFP-1010, AVES labs)<br>The secondary antibodies were Alexa conjugated (Jackson ImmunoResearch Laboratories).
Validation	Antibody staining was validated for non-specific binding through no primary antibody controls. When applicable, the specific references for the listed antibodies are provided in Materials and Methods.

Animals and other research organisms

Policy information about studies involving animals; ARRIVE guidelines recommended for reporting animal research, and Sex and Gender in Research

Laboratory animals	Wild-type fish were AB (Oregon). Transgenic lines were Tg(smyhc1:LY-Tomato^i261 59, Tg(smyhc1:egfp)^i104 58, and Tg(-2.2mylz2:EGFP)^i135 45.
Wild animals	no wild animals.
Reporting on sex	Each experiment has males and females aggregated in each sample.
Field-collected samples	No filed-collected samples.
Ethics oversight	Experimental research on animals has been approved by the cantonal veterinary office of Fribourg.

Note that full information on the approval of the study protocol must also be provided in the manuscript.

Plants

Seed stocks

Report on the source of all seed stocks or other plant material used. If applicable, state the seed stock centre and catalogue number. If plant specimens were collected from the field, describe the collection location, date and sampling procedures.

Novel plant genotypes

Describe the methods by which all novel plant genotypes were produced. This includes those generated by transgenic approaches, gene editing, chemical/radiation-based mutagenesis and hybridization. For transgenic lines, describe the transformation method, the number of independent lines analyzed and the generation upon which experiments were performed. For gene-edited lines, describe the editor used, the endogenous sequence targeted for editing, the targeting guide RNA sequence (if applicable) and how the editor was applied.

Authentication

Describe any authentication procedures for each seed stock used or novel genotype generated. Describe any experiments used to assess the effect of a mutation and, where applicable, how potential secondary effects (e.g. second site T-DNA insertions, mosiacism, off-target gene editing) were examined.
